# A protective cGAMP-mediated anti-tumor immune response can proceed without LRRC8/VRAC channels

**DOI:** 10.1016/j.jbc.2025.111060

**Published:** 2025-12-17

**Authors:** Fabian M.B. Thöne, Maya M. Polovitskaya, Uta E. Höpken, Armin Rehm, Thomas J. Jentsch

**Affiliations:** 1 Leibniz-Forschungsinstitut für Molekulare Pharmakologie (FMP), Berlin, Germany; 2 Graduate Program of the Freie Universität, Berlin, Germany; 3 Translational Tumor Immunology, Max-Delbrück-Centrum für Molekulare Medizin (MDC), Berlin, Germany; 4 Neurocure Cluster of Excellence, Charité Universitätsmedizin, Berlin, Germany

**Keywords:** cancer biology, cyclic nucleotide, tumor immunology, innate immunity, chloride channel, VSOR, SWELL1, adaptive immune response, cytokines, cell proliferation

## Abstract

The volume-regulated anion channel (VRAC) is a hetero-hexamer composed of LRRC8A and any of the four other LRRC8 paralogs (LRRC8B–E). Depending on their subunit composition, VRACs not only transport chloride, but also a range of organic substrates including 2′3′-cGAMP (cGAMP). Transfer of this immunomodulator from tumor to host cells is critical for antitumor immunity. Whether this process depends on VRAC *in vivo* remains incompletely understood. To address this issue, we studied subcutaneous MC38 and B16-F10 tumors in syngeneic mice. Enhanced growth of MC38 tumors lacking cGAMP production confirmed the importance of tumor-produced cGAMP. The impact of VRAC-mediated cGAMP-efflux from tumor cells and its uptake into cells of the tumor microenvironment was investigated using LRRC8A-deficient tumor cells and recipient mice with selective LRRC8 subunit disruptions, respectively. Changed serum cytokines indicated moderate immunomodulatory effects of VRAC-mediated cGAMP export from MC38 tumors. However, tumor growth and the cGAMP-mediated antitumor immune response were independent of both tumor- and host-expressed VRAC. Disruption of any of the non-essential subunits, LRRC8B–LRRC8E, had no discernible effect on T or B cell development in mice. While tumor-produced cGAMP markedly suppresses tumor growth, transport of this immunomodulator to the tumor environment primarily involves transporters distinct from VRAC.

The immune response against cancer can be triggered by the detection of tumor-derived damage-associated molecular patterns (DAMPs) leading to activation of innate immune cells (1). These cells secrete cytokines and chemokines and present tumor-associated antigens to adaptive immune cells. This cellular interplay can mount a productive tumor-specific immune response. Under physiological conditions, self-DNA is prevented from acting as a DAMP by being contained in mitochondria and the nucleus. However, genome instability is a hallmark of cancer (2) and frequently leads to the formation of DNA-containing micronuclei which are prone to rupture (3). This exposes the cytosolic enzyme cyclic GMP-AMP synthase (cGAS) (4, 5, 6) to self-DNA. Upon DNA binding, cGAS synthesizes the dinucleotide 2′3′-cyclic guanosine monophosphate-adenosine monophosphate (2′3′-cGAMP, hereafter referred to as cGAMP), a second messenger (7, 8) which activates its intracellular receptor, stimulator of interferon genes (STING) (9, 10). STING activates TANK-binding kinase 1 (TBK1), which phosphorylates the transcription factor interferon regulatory factor 3 (IRF3). IRF3, together with nuclear factor kappa B, induces the expression of cytokines and triggers the production of type I interferons (11, 12) which are central for an efficient anti-tumor immune response (13).

Downregulation of tumor cell-intrinsic STING signaling, which is observed in various cancers (14, 15, 16, 17), might represent a mechanism for immune evasion. STING activation in non-cancer cells of the tumor microenvironment (TME) may counteract this immune evasion (18). In subcutaneous tumor models, cGAMP-mediated immune responses require tumor-expressed cGAS and host-expressed STING, whereas tumor-expressed STING and host-expressed cGAS are dispensable (19, 20, 21, 22, 23). This strongly suggests that the “immunotransmitter” cGAMP is transferred from cancer cells to cells in the TME.

The negative charge of cGAMP prevents diffusion across the plasma membrane (PM). Several channels and transporters were recently shown to mediate cGAMP transport across the plasma membrane: LRRC8/VRAC (24, 25), SLC19A1 (26, 27), SLC46A2 (28), P2X7R (22), ABCC1 (29) and ABCC10 (30). cGAMP can also be transferred between neighboring cells *via* gap junctions (31) or taken up through phagocytosis of cGAMP-containing cells (32), thereby bypassing the extracellular space. The predominant mode of transfer is likely context- and cell type-dependent and is largely unknown.

The volume regulated anion channel (VRAC, also known as VSOR or VSOAC), best known for its role in cell volume regulation (33), is ubiquitously expressed in vertebrate cells. VRAC channels open in response to cell swelling. The resulting release of chloride and other osmolytes leads to water efflux and cell shrinkage. Five members of the leucine-rich repeat-containing 8 family, *i*.*e*. LRRC8A, -B, -C, -D and -E, were identified as pore forming subunits of the channel (34), with LRRC8A being obligatory, but not sufficient (34, 35). LRRC8 subunits assemble to hexamers which surround a central pore (36, 37, 38). In addition to chloride, VRACs conduct various small molecules. The specificity for individual substrates is determined by VRAC’s subunit composition. Inclusion of LRRC8D stimulates the transport of the anti-cancer drug cisplatin (39) and a number of neutral and positively-charged metabolites (40), whereas LRRC8E favors negatively charged substrates (40). VRACs were recently shown to permeate cGAMP when including LRRC8C or LRRC8E subunits (24, 25). Supporting a role of VRAC in immunity, mice lacking LRRC8E exhibit increased susceptibility to HSV-1 infection (24), while LRRC8C influences the immune response to influenza virus infection and autoimmune encephalomyelitis (41). These observations suggest that VRAC-mediated cGAMP transport might also bolster the immune response against cancer. Additional support for a role of VRAC in cancer comes from studies reporting a positive correlation of LRRC8A expression with poor prognosis in several types of cancer (42, 43, 44), which, however, has been tentatively attributed to cGAMP-unrelated roles of VRAC in tumor cell proliferation and apoptosis.

Here we investigated the influence of VRAC on tumor growth and the anti-tumor immune response in subcutaneous, syngeneic mouse models. Using LRRC8A disruption in MC38 (colon adenocarcinoma) and B16-F10 cells (melanoma), we examined VRAC's role in cGAMP export from tumor cells. Mice with constitutive disruption of LRRC8C and LRRC8E, or with dendritic cell (DC)-specific LRRC8A depletion, were used to study potential effects of VRAC-mediated cGAMP import into cells of the TME. Surprisingly, our findings indicate that VRAC is dispensable for both tumor growth and cGAMP-mediated anti-tumor immune responses. However, lack of LRRC8A in MC38 tumors resulted in decreased blood serum levels of inflammatory cytokines and chemokines that are usually stimulated by cGAMP-dependent signaling. We suggest that VRAC-mediated cGAMP export from tumor cells sufficed to elicit these changes but was not large enough to significantly reduce tumor growth.

## Results

### Integrity of the cGAS-STING pathway in MC38 and B16-F10 cells

To explore the role of LRRC8/VRAC channels in tumor growth and cGAMP-mediated anti-tumor immunity, we selected the MC38 colon adenocarcinoma and B16-F10 melanoma models, which elicit different immune responses and are frequently used in related studies (19, 21, 22, 23, 45, 46). Subcutaneous implantation of these cells into syngeneic mice (C57BL/6) allows investigation of emerging tumors in the presence of an intact immune environment (47, 48, 49).

**Figure 1:**
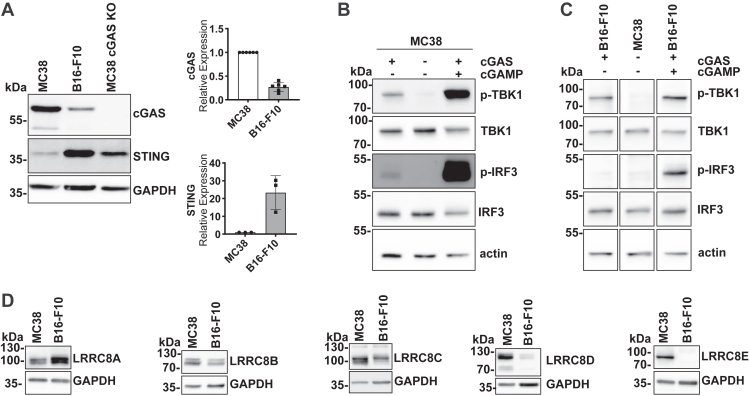
**MC38 and B16-F10 cells have a functional cGAS-STING pathway and express LRRC8 proteins**. *A*, Western blot analysis of cGAS (*n* = 6) and STING (*n* = 3) expression in MC38 and B16-F10 cells. Note the increased expression of STING in cGAS-deficient MC38 cells (*n* = 3) that were generated with CRISPR-Cas9. *B* and *C*, Western blot detection of phosphorylated STING downstream targets TBK1 and IRF3 as readouts for endogenous cGAMP production and uptake of extracellular cGAMP (*n* ≥ 4). MC38 (*B*) or B16-F10 (*C*) cells were incubated for 3 h in isotonic solution (302 mOsm/L) in the presence or absence of 10 μg/ml extracellular cGAMP. *D*, comparison of LRRC8A-E protein levels between MC38 and B16-F10 cells by Western blot (*n* = 3–4). Band intensities in *A* were quantified using ImageJ, normalized to the intensity detected in MC38 cells and presented as mean ± SD.

Both WT cell lines expressed the cGAMP-producing enzyme cGAS and the intracellular cGAMP receptor STING (Fig. 1*A*). Compared to B16-F10 cells, MC38 cells expressed more cGAS and much less STING. This suggests an inverse relationship between the two proteins, as further supported by increased STING protein levels after CRISPR-Cas9-mediated deletion of cGAS in MC38 cells (Fig. 1*A*). This might be explained by a degradation of STING following its activation by cGAMP (50). Both cell lines were able to transport cGAMP over the plasma membrane (PM) as revealed by phosphorylation of TBK1 and IRF3, downstream targets of STING, after incubation with extracellular cGAMP (10 μg/ml for 3 h in isotonic solution) (Fig. 1, *B* and *C*). Importantly, MC38 cells intrinsically produced cGAMP as revealed by background levels of TBK1 and IRF3 phosphorylation even without cGAMP addition which were absent in cGAS KO cells (Fig. 1*B*, Fig. S1*A*).

### VRAC-mediated cGAMP transport in MC38 and B16-F10 cells

Both cell lines expressed the obligatory VRAC subunit LRRC8A and the facultative subunits LRRC8B, -C and -D (Fig. 1*D*). LRRC8E could be detected in MC38, but not in B16-F-10 cells, which also expressed less LRRC8C and much less LRRC8D compared to MC38.

**Figure 2:**
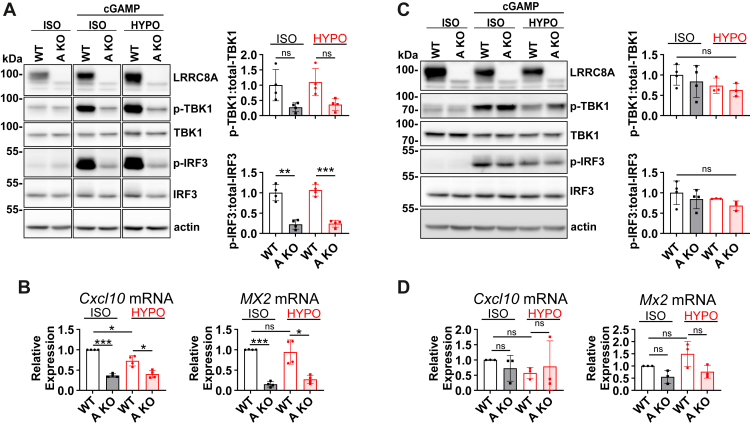
**MC38 but not B16-F10 cells use VRAC as dominant cGAMP importer**. *A*–*D*, MC38 (*A* and *B*) and B16-F10 (*C* and *D*) cells were incubated for 3 h in isotonic (302 mOsm/L) or hypotonic (217 mOsm/L) solution containing 10 μg/ml cGAMP (*n* = 3–4). Uptake of cGAMP was inferred by Western blot detection of TBK1 and IRF3 phosphorylation (*A* and *C*) and by transcript levels of *Cxcl10* and *Mx2* quantified by qRT-PCR (*B* and *D*). Data are represented as mean ± SD. Data in *panels A* and *B* were analyzed by two-way ANOVA (factors: genotype x treatment; *A* genotype: ∗∗*p* = 0.0016, *A* treatment = ns, *C* genotype = ns, *C* treatment = ns). For *panel A*, planned pairwise comparisons using Welch´s *t* test were performed. For data in *panels B* and *D*, comparisons to a normalized control value of 1 were performed using one-sample t-tests; other comparisons within the qRT-PCR data were analyzed using Welch´s *t* test after confirming normality (Shapiro-Wilk test). Benjamini-Hochberg correction was performed for multiple comparisons in *panels A*, *B* and *D*. ∗*p* < 0.05; ∗∗*p* < 0.01; ∗∗∗*p* < 0.001.

VRAC’s contribution to PM cGAMP transport was investigated using MC38 and B16-F10 cells disrupted for *Lrrc8a* (Fig. S1, *B* and *C*). To minimize clonal effects in functional assays, polyclonal cell lines comprising five and seven individual clones were produced. Additionally, a guide RNA lacking a target sequence in the mouse genome was employed to generate polyclonal MC38 and B16-F10 cell lines comprising seven and eight individual clones, respectively. These were used as WT controls throughout the study. Although intracellular production of cGAMP and its negative charge favor efflux over influx, we rather assessed cGAMP uptake because effects on downstream cGAMP targets provide sensitive, albeit indirect and non-linear, measures of transport rates. Moreover, uptake experiments allow to impose much larger cGAMP gradients than efflux assays. Measuring uptake rather than efflux is adequate, as the direction of transport through channels solely depends on the electrochemical gradient of the respective substrate. In MC38 cells, *Lrrc8a* disruption almost completely abolished the phosphorylation of TBK1 and IRF3 elicited by extracellular cGAMP (10 μg/ml for 3 h in isotonic solution) (Fig. 2*A*). It also decreased the transcription of the interferon-stimulated genes (ISGs) *Cxcl10* and *Mx2*, which are known to be inducible by cGAMP (Fig. 2*B*). We conclude that VRAC is the dominant PM cGAMP transporter of MC38 cells. Although VRAC opening by hypotonic cell swelling enhances cGAMP transport in other cells (24, 25, 51), adding cGAMP in hypotonic solution (217 mOsm/L) failed to further increase TBK1 and IRF3 phosphorylation (Fig. 2*A*) or *Cxcl10* and *Mx2* transcript levels (Fig. 2*B*). cGAMP uptake under isotonic conditions appears sufficient to saturate the downstream signaling cascade under present conditions.

The low expression of cGAMP transport-stimulating subunits LRRC8C and LRRC8E in B16-F10 cells (Fig. 1*D*) suggested minor VRAC-mediated cGAMP transport in these cells. Indeed, deletion of LRRC8A in B16-F10 cells reduced neither the cGAMP-induced phosphorylation of TBK1 or IRF3 (Fig. 2*C*) nor the transcription of *Cxcl10* and *Mx2* (Fig. 2*D*), irrespective of the osmolarity of the uptake solution. Hence, cGAMP uptake by B16-F10 cells occurs primarily through pathways distinct from VRAC.

Taken together, MC38 cells produce cGAMP endogenously and use VRAC as the dominant cGAMP transporter. This renders them suitable for studying the effects of VRAC-mediated cGAMP export in tumor models. B16-F10 cells, on the other hand, virtually lack VRAC-mediated cGAMP transport and can serve as a control to investigate cGAMP-independent effects of VRAC.

### Tumor-derived cGAMP activates the immune response against MC38 tumors

Before examining potential roles of VRAC in the cGAMP-mediated anti-tumor immune response, we used a cGAS-deficient MC38 cell line (Fig. S1*A*) to validate that tumor-produced cGAMP affects cancer growth *in vivo*. Although cGAS was recently reported to be required for VRAC activation by tumor necrosis factor (TNF) and cell swelling (51), typical hypotonicity-induced VRAC currents were still present in cGAS-deficient MC38 cells (Fig. S1*D*) while they were lost upon *Lrrc8a* disruption.

**Figure 3:**
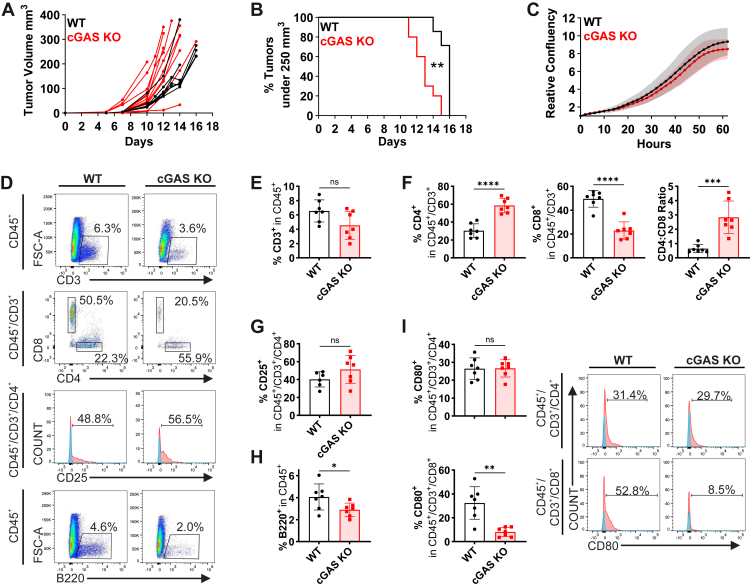
**cGAS deficiency in MC38 tumors accelerates tumor growth and changes T and B cell infiltration.***A* and *B*, WT (*n* = 7) or cGAS-deficient (*n* = 11) MC38 cells were subcutaneously injected into recipient WT mice. Tumor volumes were plotted as tumor growth curves (*A*) and as Kaplan-Meier curves using a volume of 250 mm^3^ as cutoff (*B*). ∗∗*p* < 0.01 (log-rank Mantel-Cox test). *C*, *in-vitro* proliferation of cGAS-deficient MC38 cells (*n* = 4). *D*, representative flow cytometry plots for the populations shown in *E*–*H*. *E*–*H*, flow cytometric analysis of tumor-infiltrating T and B cells shown as percentage of respective parent gate (*n* = 6–7). *I*, flow cytometric quantification of CD80-positive T cells (*n* = 7) with representative flow cytometry plots. Fluorescence minus one (FMO) control staining (*blue*) was used to define the CD25- and CD80-positive gates (*D* and *I*). Note that the control group (WT MC38 tumors in WT mice) was partially shared with experiments involving *Lrrc8c*^−/−^ mice; therefore, portions of the data presented here are also included in Figures 6 and S5. Data in *E*–*I* are represented as mean ± SD. Normality was confirmed for all data except for the CD4:CD8 ratio (Shapiro-Wilk test). Normally distributed data were analyzed using unpaired Welch´s *t* test, while Mann-Whitney U test was used for non-normally distributed data. ∗*p* < 0.05; ∗∗*p* < 0.01; ∗∗∗*p* < 0.001; ∗∗∗*p* < 0.0001.

We subcutaneously transplanted WT or cGAS-deficient MC38 cells into recipient mice, measured the diameters of emerging tumors, and calculated tumor volumes (52). Tumor growth curves (Fig. 3*A*) and Kaplan-Mayer plots (Figs. 3*B* & S2*A*) revealed accelerated tumor growth in the absence of cGAMP-producing cGAS. Crucially, this effect did not result from altered proliferation of KO cells as assessed *in vitro* (Fig. 3*C*). The cGAMP-mediated immune response against MC38 tumors is mainly driven by CD8^+^ cytotoxic T cells (53, 54). Accordingly, flow cytometric analysis of T cells (Fig. 3, *D*–*G*) revealed a decreased frequency of CD8^+^ cells in cGAS-deficient tumors. There was also a decreased percentage of B220^+^ B cells (Fig. 3*H*), while several myeloid cell populations remained unchanged (Fig. S2*B*). The effects on T and B cells were tumor-specific as they were absent in spleens of the same animals (Fig. S2*C*). cGAMP uptake by T cells induces p53 signaling resulting in increased surface expression of CD80 (41). Indeed, the percentage of CD80^+^ cells within the CD8^+^ population was markedly reduced in cGAS-deficient tumors (Fig. 3*I*). Since this effect was also present in spleens of the same animals (Fig. S2*D*) it is likely indirect.

### MC38-expressed VRAC does not impact tumor growth but mildly affects the anti-tumor immune response

**Figure 4:**
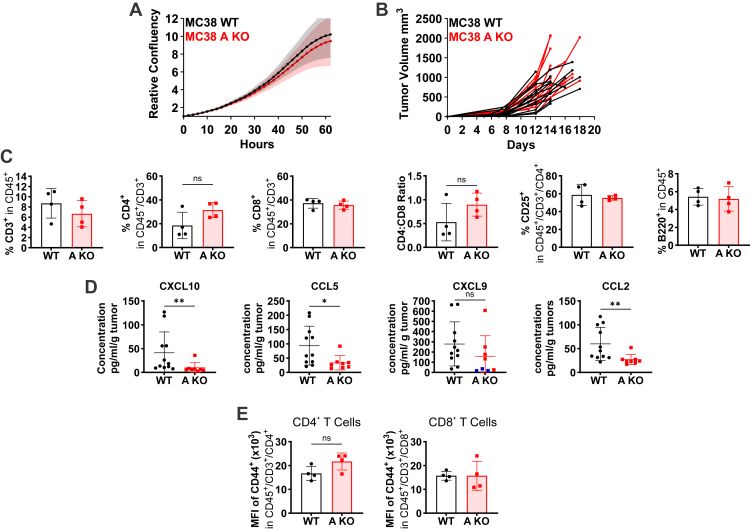
**VRAC channels are dispensable for MC38 tumor growth but contribute to immunomodulation**. *A*, *in vitro* proliferation of LRRC8A-deficient MC38 cells (*n* = 6). *B*, WT and LRRC8A-deficient MC38 cells were subcutaneously injected into recipient WT mice (*n* = 12). Tumor volumes were plotted as tumor growth curves. *C*, flow cytometric analysis of tumor-infiltrating T and B cells in MC38 tumors shown as percentage of respective parent gate (*n* = 4). *D*, blood serum chemokine and cytokine concentrations in mice bearing WT (*n* = 11) or LRRC8A-deficient (*n* = 8) MC38 tumors. Blood serum was obtained at the day of tumor resection and subjected to multiplex cytokine assay. Serum cytokine concentrations from individual mice were normalized to the weight of the corresponding tumor. Datapoints below the detection limit of the assay were assigned the lowest detected value and are shown in *blue. E*, flow cytometric analysis of tumor-infiltrating T cell activation quantified as median fluorescence intensity (MFI) of CD44 (*n* = 4). Data are represented as mean ± SD. Normality was confirmed for all data in panels *C* and *E*, but rejected for all data in *panel D* (Shapiro-Wilk test). Normally distributed data were analyzed using unpaired Welch´s *t* test, while Mann-Whitney U test was used for non-normally distributed data. ∗*p* < 0.05; ∗∗*p* < 0.01.

MC38 cells were used to assess the role of VRAC-dependent cGAMP transport in tumor growth because of their intrinsic cGAMP production (Fig. 1*B*) and the predominant role of VRAC in cGAMP transport (Fig. 2, *A* and *B*). Crucially, disruption of LRRC8A did not affect MC38 proliferation *in vitro* (Fig. 4*A*). We transplanted WT and LRRC8A-deficient MC38 cells into recipient mice and monitored tumor growth. In contrast to cGAS-deficient tumors, neither changes in tumor growth (Fig. 4*B*) nor statistically significant effects in T and B cell subsets were observed (Fig. 4*C*). However, LRRC8A-deficient tumors showed an increased percentage of CD11b^+^/CD11c^+^ cells (Fig. S3*A*) and a non-significant increase in the frequency of CD4^+^ T cells (Fig. 4*C*). Measurements of serum parameters also suggested LRRC8A-dependent effects of cGAMP. Production of pro-inflammatory chemokines and cytokines CXCL10, CCL5, CXCL9 and CCL2 is stimulated by STING activation (24, 27, 55, 56, 57, 58, 59, 60). Except for CXCL9, they were significantly reduced in sera of mice bearing LRRC8A-deficient tumors (Fig. 4*D*). Importantly, IL-1α, IL-23, and IL-2, cytokines independent of STING signaling, were not affected (Fig. S3*B*). Indicative of an altered myeloid cell regulation, the hematopoietic growth factor G-CSF was reduced when tumors lacked LRRC8A (Fig. S3*C*). Activation of tumor-infiltrating T cells was unchanged as surface expression of the activation marker CD44 was not affected by MC38-expressed LRRC8A (Figs. 4*E* & S3*D*). We conclude that VRAC-mediated cGAMP export from MC38 tumors modulates ISG expression but is too weak to reduce tumor growth.

**Figure 5:**
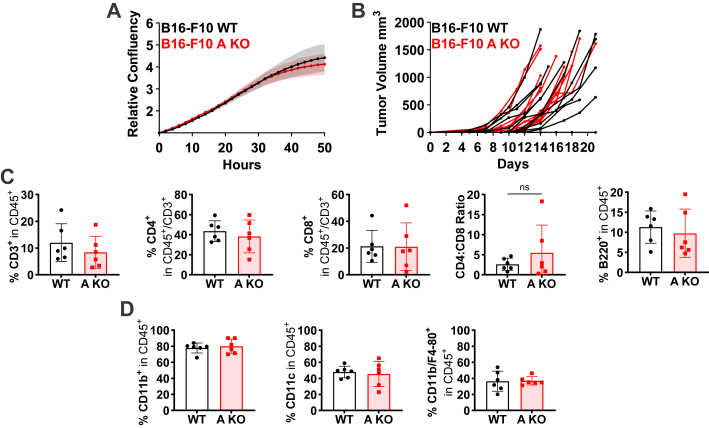
**VRAC channels are dispensable for B16-F10 tumor growth**. *A*, *in vitro* proliferation of LRRC8A-deficient B16-F10 cells (*n* = 3). *B*, WT and LRRC8A-deficient B16-F10 cells were subcutaneously injected into recipient WT mice (*n* = 11 and 12). Tumor volumes were plotted as tumor growth curves. *C* and *D*, flow cytometric analysis of tumor-infiltrating T and B cells (*C*) and myeloid cells (*D*) shown as percentage of respective parent gate (*n* = 6). Data are represented as mean ± SD. Normality was confirmed for all data in *panels C* and *D* and unpaired Welch´s *t* test was used for analysis. ∗*p* < 0.05.

VRACs have been proposed to promote cancer cell proliferation in a cGAMP-independent manner (42, 43, 44). However, LRRC8A-deficient MC38 cells showed normal proliferation *in vitro*, and tumor growth was unaltered. As VRAC’s influence on proliferation might be cell type-specific, we extended our investigation to B16-F10 cells even though they show negligible VRAC-dependent cGAMP uptake. Similar to MC38 cells, *Lrrc8a* disruption neither affected their proliferation *in vitro* (Fig. 5*A*), nor tumor growth *in vivo* (Fig. 5*B*). The frequencies of T and B cells (Fig. 5*C*) and several myeloid cell subsets (Fig. 5*D*) within tumors were likewise unaltered.

We conclude that tumor cell-expressed VRACs are dispensable for proliferation *in vitro* and have no detectable effects on tumor growth and immune cell infiltration *in vivo*. Nonetheless, alterations in serum chemokines and cytokines indicated an immunomodulatory potential of VRAC-mediated cGAMP transport.

### Host-expressed VRAC channels are dispensable for tumor growth and the cGAMP-mediated anti-tumor immune response

As VRAC channels transport substrates passively according to their electrochemical gradient, they may be involved in both cGAMP efflux from tumor cells and influx into cells of the TME. The latter aspect was investigated using recipient mice in which we had disrupted LRRC8 subunits either constitutively or only in specific cell populations. Whereas *Lrrc8a*^−/−^ mice cannot be used as tumor hosts because of their high premature mortality (61), mice lacking any of the other subunits are viable. *Lrrc8b*^*−/−*^, *Lrrc8c*^−/−^ and *Lrrc8e*^−/−^ mice lack immediately obvious phenotypes (24, 62) while *Lrrc8d*^−/−^ mice develop proximal tubular degeneration (62). Before using these animals in tumor experiments, we assessed their immune cell populations in peripheral blood, spleen, and lymph nodes at an age between 8 to 12 weeks. No significant differences in frequencies of T and B cells or any other tested immune cell population were observed (Fig. S4, *A*–*C*) and spleen weights were not altered (Fig. S4*B*).

**Figure 6:**
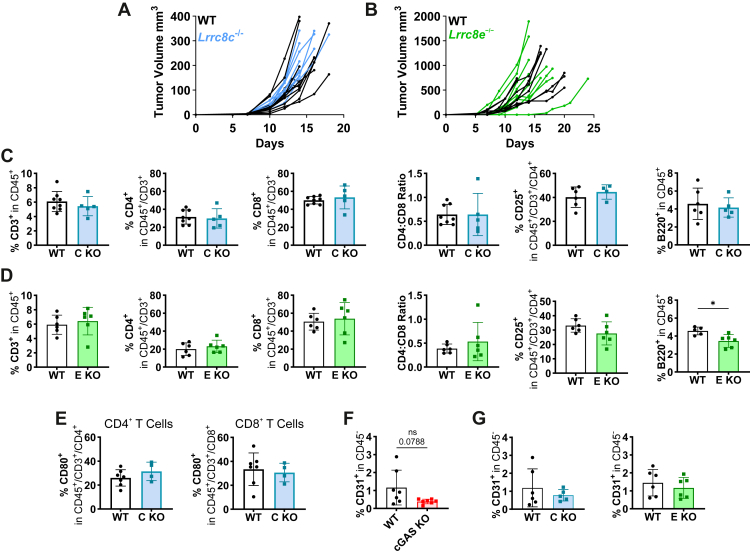
**Lack of LRRC8C or LRRC8E in recipient mice does not influence tumor growth or immune cell infiltration**. *A* and *B*, MC38 cells were subcutaneously injected into *Lrrc8c*^−/−^ (*n* = 12 and *n* = 9 for WT and *Lrrc8c*^−/−^ mice, respectively) (*A*) or *Lrrc8e*^−/−^ (*n* = 9) (*B*) recipient mice. Tumor volumes were plotted as tumor growth curves. *C* and *D*, flow cytometric analysis of tumor-infiltrating T and B cells in MC38 tumors from *Lrrc8c*^−/−^ (C KO; *n* = 5–8) (*C*) or *Lrrc8e*^−/−^ (E KO; *n* = 5–6) (*D*) mice. *E*, flow cytometric quantification of CD80-positive T cells in MC38 tumors from WT (*n* = 7) or *Lrrc8c*^−/−^ (*n* = 4) mice. *F* and *G*, flow cytometric quantification of CD31-positive endothelial cells in the CD45-negative population within MC38 tumors. Frequency of endothelial cells was compared between WT and cGAS-deficient MC38 tumors (*n* = 7) (*F*) or between WT MC38 tumors from WT (*n* = 6) and *Lrrc8c*^−/−^ (*n* = 5) or *Lrrc8e*^−/−^ (n = 6) recipient mice (*G*). Flow cytometry data is shown as percentage of parent gate and represented as mean ± SD. Normality was confirmed for all data in *C*–*G* and unpaired Welch´s *t* test was used for analysis. ∗*p* < 0.05. Note that the control group for experiments with *Lrrc8c*^−/−^ animals (WT MC38 tumors in WT mice) was partially shared with experiments involving cGAS KO tumors; therefore, portions of the data presented in Figure 6*F* also appear in Figure 6*G* as well as in Figures 3 and S2.

We transplanted WT MC38 cells into mice lacking either LRRC8C or LRRC8E, subunits which enhance VRAC’s ability to transport cGAMP (24, 25), but observed no differences in tumor growth (Fig. 6, *A* and *B*). Except for a small reduction of B cells in *Lrrc8e*^−/−^ mice, frequencies of lymphocytes (Fig. 6, *C* and *D*) and myeloid cells (Fig. S5, *A* and *B*) were unchanged. CD80 surface expression, which was reduced in CD8^+^ T cells when tumors lacked cGAS (Fig. 3*I*), was unaltered in tumor-infiltrating T cells from *Lrrc8c*^−/−^ mice (Fig. 6*E*).

STING activation in tumor-associated endothelial cells induces vascular remodeling and anti-tumor immunity (46) which depends on the transfer of tumor-derived cGAMP into endothelial cells (63). In line with these reports, we observed a trend towards a reduced frequency of CD45^-^/CD31^+^ endothelial cells in cGAS-deficient MC38 tumors (Fig. 6*F*). However, the percentage of CD45^−^/CD31^+^ cells was unchanged in *Lrrc8c*^−/−^ and *Lrrc8e*^−/−^ mice (Fig. 6*G*), despite the importance of LRRC8C for cGAMP uptake by endothelial cell lines (25, 63).

**Figure 7:**
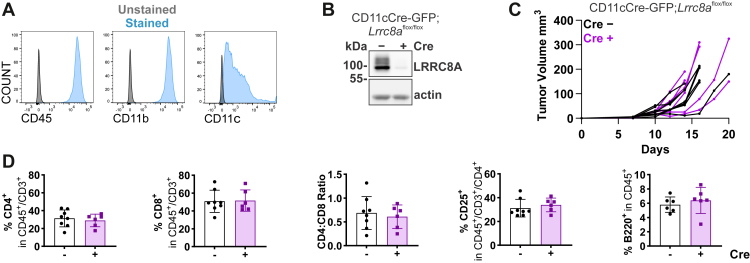
**Dendritic cell-expressed VRAC channels do not influence tumor growth or immune cell infiltration**. *A*, cellular identity of bone marrow-derived dendritic cells (BMDCs) from CD11cCre-GFP;*Lrrc8a*^flox/flox^ mice was confirmed by detection of characteristic surface markers using flow cytometry. *B*, LRRC8A knockout in BMDCs was confirmed by a Western blot. *C*, MC38 cells were subcutaneously injected into CD11cCre-GFP^+/−^;*Lrrc8a*^flox/flox^ (*n* = 8) or *Lrrc8a*^flox/flox^ (*n* = 6) recipient mice. Tumor volumes were plotted as tumor growth curves. *D*, flow cytometric analysis of tumor-infiltrating T and B cells in MC38 tumors from CD11cCre-GFP;*Lrrc8a*^flox/flox^ mice (*n* = 6–8). Data are represented as mean ± SD.

Constitutive disruption of non-essential LRRC8 isoforms has the advantage that all cells lack the respective subunit, while the remaining LRRC8 heteromers can still regulate cell volume. However, cGAMP transport will probably be retained to some degree. As an additional approach, we therefore deleted VRAC completely in dendritic cells (DCs) by crossing *Lrrc8a*^lox/lox^ mice (64) with CD11cCre-GFP animals (65). We chose DCs as they react to tumor-derived cGAMP (20, 21) and since cGAMP enhances cross-presentation of tumor-associated antigens. This leads to a CD8^+^ T cell response (66), which was changed in cGAS KO MC38 tumors (Fig. 3*F*). To confirm efficient deletion of LRRC8A in dendritic cells, we produced bone marrow-derived dendritic cells (BMDCs) from CD11cCre-GFP;*Lrrc8a*^lox/lox^ mice, validated their identity by flow cytometry (Fig. 7*A*), and confirmed efficient LRRC8A deletion by Western blot (Fig. 7*B*). Disruption of *Lrrc8a* likely extended more broadly to the myeloid compartments (Fig. S5*C*). Effects of *CD11c*Cre-driven *Lrrc8a* disruption were neither observed on MC38 tumor growth (Fig. 7*C*), nor on tumor-infiltrating immune cell populations (Figs. 7*D* & S5*D*).

## Discussion

Transfer of the immunomodulator cGAMP from cancer cells to cells within the tumor microenvironment (TME) has been reported to induce an anti-tumor immune response, resulting in tumor suppression (19, 20, 21, 22, 23). The recent identification of volume-regulated anion channels (VRACs) as conduits for cGAMP suggests that VRACs may restrict tumor growth by facilitating cGAMP transfer. On the other hand, VRACs might enhance tumor growth by stimulating cell proliferation and migration. We used subcutaneous tumor models in which VRAC/LRRC8 subunits were disrupted in either tumor cells or recipient mice. No effects on tumor cell proliferation *in vitro*, nor on tumor growth *in vivo* were observed. Although analysis of tumor-infiltrating immune cells revealed no differences either, changes in serum chemokines and cytokines suggested a modest effect of VRAC-mediated cGAMP export from MC38 cells. We conclude that VRAC is not the major pathway for cGAMP transmission, at least in our model.

VRAC-dependent cell volume regulation has long been considered important for cell division, proliferation and migration (67, 68). Accordingly, high LRRC8A expression was linked with poor prognosis in several cancer types (42, 43, 44, 69), although databases also reveal inverse correlations for other tumors (45, 69). Consistent with a comprehensive *in vitro* study (70), *Lrrc8a* disruption neither affected the proliferation of MC38 or B16-F10 cells *in vitro*, nor tumor growth *in vivo*. In contrast to many other studies, our experiments were performed with polyclonal cell lines to avoid VRAC-unrelated clonal variations. However, these results do not exclude a role of LRRC8 in cancer pathology in general. Indeed, *Lrrc8a* knockdown in MDA-MB-231 breast cancer cells had no effect on tumor growth in immunodeficient mice but reduced metastasis, likely by decreasing tumor cell migration (71). This may become apparent only when cells migrate in confined spaces like those encountered in tissues (71).

Our work focused primarily on the potential role of VRAC-mediated cGAMP transport on tumor growth. We first confirmed a crucial role of tumor-produced cGAMP in MC38 tumor suppression and identified VRAC as the major cGAMP exporter of these cells. Surprisingly, however, VRAC disruption in MC38 cells had no significant effect on tumor growth and immune cell infiltration *in vivo*. Taken together, these results suggest that other mechanisms of cGAMP-efflux from tumor cells are dominant in our setting. Excellent candidates are gap junctions, which do not connect the extracellular space with the cytosol and are therefore not addressed by our cGAMP uptake assay. A significant effect on serum inflammatory chemokines and cytokines suggested that parallel VRAC-mediated cGAMP exit plays an additional, modulatory role. Since we neither observed an effect on tumor growth when LRRC8 subunits were disrupted in recipient mice, our work *prima facie* suggests that VRAC does not affect tumor growth.

By contrast, important effects of host cell-expressed VRAC on immune responses were seen in other settings, such as viral infections (24, 41) and the growth of irradiated tumors (45). Disruption of *Lrrc8e* in recipient mice worsened infection by HSV-1 virus, which is associated with markedly increased cellular cGAMP levels (24). While our manuscript was in preparation, a study by Cao and coworkers (45) reporting effects of LRRC8 disruption in recipient mice, but not in MC38 tumors, was released. The authors investigated effects of radiation therapy, a procedure inducing DNA damage and thereby increasing cGAMP production (72). Of note, the curative effect of ionizing radiation has previously been reported to depend strongly on extracellular cGAMP in a subcutaneous tumor model (21). For non-irradiated tumors, the results of Cao and coworkers are consistent with those reported here. In particular, no effect of dendritic cell-specific *Lrrc8a* disruption and no enhanced tumor growth in *Lrrc8c*^−/−^ recipient mice was observed (45). In the latter case, contrasting with our study, they paradoxically even observed a mild decrease in tumor growth. The curative effect of radiation therapy depended on tumor-expressed cGAS and was strongly reduced in *Lrrc8c*^−/−^ mice or in mice carrying DC and T cell specific *Lrrc8a* disruptions. While it is currently unknown whether VRAC-disruption in tumor cells would also impact the efficacy of irradiation therapy, Cao *et al*. (45) conclusively show a pivotal role of VRAC-dependent cGAMP uptake into T cells after irradiation.

To explain the role of VRAC in irradiated tumors or virus-infected cells, we suggest that under these conditions extracellular cGAMP levels are highly increased. The immunotransmitter cGAMP can then diffuse from tumor cells to target cells beyond those connected by gap junctions. In viral infection, VRAC’s contribution to immunoregulation may be increased by an inhibition of gap junctions (73). Moreover, VRACs of recipient T cells may be activated by ATP, T cell receptor stimulation and reactive oxygen species (ROS) as proposed for the tumor setting (45).

Intratumoral administration of non-hydrolyzable cGAMP analogs is another possibility to activate STING in tumor therapy. Like cGAMP itself, these analogs might be transported by VRACs as recently shown for ADU-S100 (25). cGAMP analogs had strong curative effects in murine tumor models (59, 74) and are under clinical investigation in humans (NCT03010176, NCT04220866, NCT04020185 and NCT05846659). However, therapies relying on STING activation are double-edged swords. While STING activation in myeloid and endothelial cells has tumor-suppressive effects (28, 75, 76, 77, 78), its overactivation in T cells may rather decrease the anti-tumor immune response (74, 79). Expression profiling of the cGAMP-transport-stimulating subunits LRRC8C and LRRC8E in different recipient cells may reveal cell type-specific routes for cGAMP import. Combining radiation or cGAMP analog treatments with subunit-specific modulation of VRAC activity might limit unwanted effects while amplifying the intended therapeutic response.

## Conclusion

Our work indicates that VRAC has no general role in cell proliferation or tumor growth and that disruption of non-essential LRRC8 subunits lacks effects on immune cell development and homeostasis. LRRC8-dependent changes of serum chemokines and cytokines in tumor-bearing mice suggest moderate VRAC-dependent immune responses which are markedly increased by infectious diseases (24, 41) or therapeutic intervention such as irradiation (45) that increase cGAMP production. It remains to be seen whether these findings can be extended to other tumor types and other mechanisms of cGAMP transfer. Modulating cGAMP transfer may be useful to enhance immune responses against cancer and other pathologies that benefit from immune activation. Conversely, VRAC-inhibition may be leveraged to treat autoimmune disorders associated with elevated cGAMP signaling.

## Experimental procedures

### Mice

All animal experiments were performed in compliance with the institutional guidelines of the Max Delbrück Center for Molecular Medicine (MDC) and the Leibniz-Forschungsinstitut für Molekulare Pharmakologie (FMP) and have been approved by the Berlin authorities (LAGeSo). Mice were housed at the MDC animal facility under standard conditions. Mice with constitutive disruptions of *Lrrc8b* (62), *Lrrc8c* (62), *Lrrc8d* (62) or *Lrrc8e* (24) have been described previously. CD11cCre-GFP;*Lrrc8a*^flox/flox^ mice, which have a dendritic cell-specific knockout of *Lrrc8a*, were generated by crossing CD11cCre-GFP (C57BL/6J-Tg(Itgax-cre,-EGFP)4097Ach/J) (65) animals with *Lrrc8a*^flox/flox^ (Lrrc8atm2c(EUCOMM)Hmgu) (64) mice. They were kept homozygously floxed and heterozygous for Cre. C57BL/6J-Tg(Itgax-cre,-EGFP)4097Ach/J mice were obtained from Jackson Laboratories. All mice had a C57Bl/6 background and were used at an age of 8 to 15 weeks.

### Cell lines and culture conditions

MC38 (provided by T. Blankenstein, MDC, Berlin) and B16-F10 (ATCC, #CRL6475) cells were cultured in RPMI 1640 (PAN Biotech, P04–16500) or DMEM (PAN Biotech, P04–03550), respectively. Medium was supplemented with 10% fetal calf serum (FCS) (PAN Biotech, P40–37500), 100 U/ml penicillin (PAN Biotech, P06–07100) and 0.1 mg/ml streptomycin (PAN Biotech, P06–07100) and is hereafter referred to as complete culture medium. Cells were maintained under standard culture conditions in the presence of 5% CO_2_ at 37 °C. Both cell lines tested negative for *mycoplasma* contamination.

### Generation of knockout cell lines with CRISPR-Cas9

Target sequences specific for *Lrrc8a and Cgas* (Table S1) were designed with the Benchling software and cloned into PX458 vector (Addgene, 48138) expressing GFP, Cas9 and a single guide RNA (gRNA). Cells were transfected using Lipofectamine 2000 (Invitrogen, 11668019), and single-cell FACS of GFP-positive cells was performed with a FACSAria II or FACSAria III (BD Bioscience) device. Gene editing in arising monoclonal cell lines was validated by Sanger sequencing using the ICE deconvolution algorithm (Synthego). Polyclonal cell lines were produced by combining equal proportions of several knockout clones. Knockout was reconfirmed by Western blot. Polyclonal WT control cell lines consisting of 7 and 8 individual clones for MC38 and B16-F10 cells, respectively, were produced as described above using a gRNA without a target sequence in the mouse genome (Table S1). These cell lines were used as WT control throughout the study.

### cGAMP import assay

0.7 x 10^6^ MC38 or B16-F10 cells were seeded in a 6-well format 1 day before treatment. Culture medium was removed and cells were incubated for 3 h in isotonic (302 mOsm/L; 85 mM mannitol, 90 mM NaCl, 1 mM MgCl_2_, 2 mM CaCl_2,_ 4 mM KCl, 10 mM glucose, 10 mM HEPES pH 7.4) or hypotonic (217 mOsm/L; 90 mM NaCl, 1 mM MgCl_2_, 2 mM CaCl_2,_ 4 mM KCl, 10 mM glucose, 10 mM HEPES pH 7.4) solution containing 10 μg/ml 2′3′-cGAMP (InvivoGen, tlrl-nacga23–02). Cells were cryopreserved at −80 °C until further use.

### Western blot

Cells were lysed in RIPA buffer (50 mM Tris-HCl pH 8, 150 mM NaCl, 1% Triton-X100 and 0.5% deoxycholate) supplemented with protease inhibitors cOmplete (Merck, 11836145001) and Pefabloc (Roth, A154.3). When phosphorylated proteins were detected, additional phosphatase inhibitors were added: 10 mM pyrophosphate, 10 mM glycerophosphate, 50 mM NaF and 1.5 mM Na_3_VO_4_. Lysates were sonicated and centrifuged at 16,000 x g for 10 min at 4 °C. Protein concentrations were determined by BCA assay (Thermo Fisher, A55860). Proteins were treated with reducing Laemmli buffer (50 mM Tris pH 6.8, 0.1% bromphenol blue, 2% SDS, 5% β-mercaptoethanol and 10% glycerol) for 5 min at 80 °C or for 20 min at 55 °C when detecting membrane proteins. Equal amounts of protein were separated by SDS-PAGE on a 7.5% polyacrylamide gel and transferred to a 0.45 μm nitrocellulose membrane (Cytiva, 10600002) using the Mini Trans-Blot Electrophoretic Transfer Cell system (Bio-Rad, 1703930). Membranes were blocked for 45 min with 5% (m/v) non-fat dry milk and incubated overnight at 4 °C with primary antibodies followed by incubation at room temperature for 1 h with a peroxidase-coupled secondary antibody (antibodies and concentrations are listed in Table S2). Detection was performed with either the SuperSignal West Pico Kit (Thermo Fischer, 34580) or the Western BLoT HYPER HRP substrate (TaKaRa, T7103 A) using the Chemi-Smart 5000 CCD camera (PeqLab) and ChemiCapt 5000 software (PeqLab). Signals were quantified using ImageJ.

Specificity of antibodies detecting LRRC8 proteins as well as cGAS was validated using MC38 (LRRC8A, -C, -D, -E, cGAS), B16-F10 (LRRC8A) or HEK293 T (LRRC8A-E) KO cell lines, while the STING-specific antibody was validated with BMDMs from STING^gt/gt^ mice (80). Antibodies against p-TBK1 and p-IRF3 were validated by detection of increased levels of phosphorylated target proteins in MC38 and B16-F10 cells after stimulation with cGAMP. Antibodies against total TBK1 and IRF3 proteins as well as actin and GAPDH were validated by the respective manufacturer. All secondary antibodies were validated against control membranes not treated with the respective primary antibody. Detailed information on antibodies is given in the Supporting Information Table S2.

### Quantitative RT-PCR

Total RNA was extracted using the NucleoSpin RNA kit (Macherey-Nagel, 740955). Total cDNA was produced using Superscript II Reverse Transcriptase (Invitrogen, 18064014) and random primers (Invitrogen, 48190011) following the manufacturer´s instructions. Quantitative RT-PCR (qRT-PCR) was performed with the Power SYBR Green PCR Master Mix (Applied Biosystems, 4367659) on the Step One real-time PCR system (Applied Biosystems, 4376592) using primers listed in Table S1. Relative expression was calculated with the delta-delta-CT algorithm and normalized to the expression of β-actin.

### Electrophysiology

Cells were seeded onto gelatin-coated coverslips 4 to 6 h before recording. VRAC currents were recorded in the whole-cell configuration at 23 °C using an EPC-10 patch-clamp amplifier and PatchMaster v2x90.3 software (HEKA Elektronik). Signal was sampled at 5 kHz and filtered with a lowpass Bessel filter at 2.9 kHz during acquisition. Voltage was held at −30 mV between sweeps. To monitor current activation and to assess steady-state current densities, 600 ms voltage ramps from −100 to +100 mV were applied every 10 s preceded by a 200-ms step at −80 mV. Once steady-state activation was reached, a voltage step protocol was applied: voltage was held at values between −100 mV and +120 mV in 20 mV increments for 1000 ms, each step was flanked by 400 ms-long intervals at −100 mV. Patch pipette solution contained (in mM): 40 CsCl, 100 cesium methanesulfonate, 1 MgCl_2_, 5 EGTA, 4 Na_2_ATP, and 10 HEPES (pH 7.2, adjusted with CsOH 290 mOsm/kg) and had a resistance of 2 to 4 MOhm. The isotonic bath solution contained (in mM): 150 NaCl, 6 CsCl, 1 MgCl_2_, 1.5 CaCl_2_, 10 glucose, and 10 HEPES (pH 7.4, adjusted with NaOH, 320 mOsm/kg). VRAC current was elicited by a 25% hypotonic solution containing (in mM): 105 NaCl, 6 CsCl, 1 MgCl_2_, 1.5 CaCl_2_, 10 glucose, HEPES (pH 7.4, adjusted with NaOH, 240 mOsm/kg). Liquid junction potentials were not corrected for. Data analysis was performed using SciPy 1.5.2 library 71 for Python 3.8 programming language (Python Software Foundation).

### Proliferation assay

Proliferation was assessed at 37 °C and 5% CO_2_ using the IncuCyte S3 Live-Cell Analysis System (Sartorius, Essen BioScience). 0.5 x 10^4^ MC38 or 1 x 10^4^ B16-F10 cells were seeded in a 96-well format in at least triplicates and five phase contrast images of distinct areas per well were acquired. Average confluency of individual wells was calculated using the IncuCyte software package. Average confluency over at least 3 wells was calculated and normalized to the initial confluency 2 h after seeding. Each value obtained in this way represent one individual experiment. Proliferation curves were calculated from at least 3 such experiments.

### Tumor experiments

For each experimental series, cells from the same cryopreserved batch were used to ensure consistency. Only healthy-looking cells in the exponential growth phase were used. Cryopreserved cells were thawed, passaged at a 1:1 ratio the following day and further cultured for 2 days. Cells were detached using Trypsin/EDTA (Gibco, 25300054), and the enzymatic reaction was stopped with FCS-containing medium. FCS was removed by washing 3 times with PBS, and cells were resuspended in PBS. 100 μl containing 1 x 10^5^ (MC38) or 2 x 10^5^ (B16-F10) cells were injected subcutaneously into the left flank using a 26-gauge needle. Longitudinal and transversal diameters of emerging tumors were measured every 2 to 3 days with a digital caliper and the tumor volume was calculated using the modified ellipsoid formula (52):

$$Volume=\frac{1}{2}*\left(lenght*{width}^{2}\right)$$

Each experimental series consisted of at least two independent experiments with group sizes ranging from 3 to 5 animals, accompanied by an age-matched control group. All animals were between 8 and 14 weeks of age. Tumors, spleens and blood sera were harvested when tumors either reached a diameter of 15 mm or showed first signs of severe skin lesions.

### Flow cytometry

To control for tumor size as a variable affecting immune cell composition, only tumors of comparable weight between the test and the control groups were included in flow cytometric analysis (Fig. S5, *E* and *F*). Tumor fragments were enzymatically digested for 45 min at 37 °C with 0.8 mg/ml Dispase II (Gibco, 17105041), 0.2 mg/ml Collagenase P (Roche, 11249002001) and 0.33 U/ml DNAse I (Invitrogen, 18047–019). Single-cell suspensions from lymph nodes (inguinal and mesenteric) and spleens were produced by mechanical dissociation. Peripheral blood was collected from the beating heart and coagulation was inhibited using EDTA. Erythrocyte lysis was performed for blood and spleens for 2 min using red blood cell lysis buffer (Abcam, ab204733).

Single-cell suspensions were either used immediately (investigation of immune cells in naive *Lrrc8b*^−/−^ through *Lrrc8e*^−/−^ mice and experiments with LRRC8A-deficient MC38 tumors) or cryopreserved at −80 °C in FCS supplemented with 10% DMSO until further use. Fc receptor blocking was performed for 30 min at 4 °C with CD16/32-antibody. Cells were then incubated with fluorochrome-conjugated antibodies (concentrations indicated in Table S2) for 30 min on ice in the dark. 7-AAD viability dye (Invitrogen, A1310) was added, and flow cytometry data were acquired on the FACSCanto II, the LSRFortessa or the FACSymphony device (BD Bioscience). Data were analyzed using FlowJo v. 10.6.1 software.

Gating strategies for MC38 and B16-F10 tumors as well as spleens of tumor-bearing mice are shown in Figures S6–S8. Due to spectral overlap between GFP and FITC-labeled anti-CD3 antibody, gating for tumor-infiltrating T cells from CD11cCre-GFP; *Lrrc8a*^lox/lox^ mice differed from that of other experiments. GFP-positive cells were reliably excluded from the analysis by applying a stringent first gate Figure S9. Gating strategies for blood, spleens and lymph nodes of naive *Lrrc8b*^−/−^, *Lrrc8c*^−/−^, *Lrrc8d*^−/−^ and *Lrrc8e*^−/−^ mice are shown in Figures S10–S12.

### Production of bone marrow-derived dendritic cells (BMDCs)

Bone marrow was extracted from femurs and tibias of 13-week-old male mice, dissociated by repeated pipetting, and filtered through a 40 μm cell strainer. Adherent cells were discarded after 1 h of incubation, and the remaining non-adherent cells were replenished with complete RPMI 1640 culture medium supplemented with 20 ng/ml GM-CSF (Peprotech, 315–03). Cells were differentiated for 10 days under standard conditions. Every 1 to 2 days, non-adherent cells were removed, and fresh GM-CSF-containing medium was added.

### Serum cytokine detection

Blood of tumor-bearing mice was collected from the beating heart and coagulated for 10 min at 37 °C and continuous shaking. Serum was separated by centrifugation at 1000 x g for 10 min at 4 °C and stored at −80 °C until further use. Detection was performed with the 21-plex ProcataPlex (Thermo Fischer) immunoassay following the manufacturer ’s instructions using the Luminex 200 (Luminex Corporation) instrument. The following chemokines, cytokines and growth factors were analyzed: CXCL10, CCL5, IL-6, IL-12 (p70), CXCL9, IL-1β, IL-10, CCL2, IL-1α, IL-23, IL-2, IL-17A, GM-CSF, G-CSF, M-CSF, IFN-α, IFN-β, IFN-γ, IL-27, TNF- α and VEGF-A. The measurements for IL-6, IL-1β, IL-10, IL-17A, GM-CSF, M-CSF, IFN-α, IFN-β, IFN-γ, IL-27, TNF- α and VEGF-A fell near or below the detection limit of the assay and were excluded from the analysis.

### Statistical analysis

Statistical significance was defined as follows: ∗*p* < 0.05; ∗∗*p* < 0.01; ∗∗∗*p* < 0.001.

All datapoints presented in scatter plots correspond to independent biological replicates. Scatter plot data were tested for normality using Shapiro–Wilk test. Normally distributed data were analyzed using unpaired Welch´s *t* test, while non-normally distributed data were analyzed using Mann–Whitney *U* test. Western Blot data on cGAMP import (Fig. 2, *A* and *C*) were analyzed by two-way ANOVA (factors: genotype x treatment) followed by planned pairwise comparisons using Welch´s *t* test. For qRT-PCR data (Fig. 2, *B* and *D*), comparisons to a normalized control value of 1 were performed using one-sample t-tests; other comparisons within the qRT-PCR data were analyzed using Welch´s *t* test after confirming normality (Shapiro–Wilk test). Benjamini-Hochberg correction was performed for all multiple comparisons. Kaplan-Meier plots were analyzed using log-rank (Mantel-Cox) test.

## Data availability

All datasets used in this study are available from Thomas J. Jentsch.

## Supporting information

This article contains supporting information (34, 64).

## Conflict of interest

The authors declare that there are no conflicts of interests with the contents of this article.

## References

[1] Yanai H., Hangai S., Taniguchi T. (2021). Damage-associated molecular patterns and toll-like receptors in the tumor immune microenvironment. Int. Immunol..

[2] Hanahan D., Weinberg R.A. (2011). Hallmarks of cancer: the next generation. Cell.

[3] Hatch E.M., Fischer A.H., Deerinck T.J., Hetzer M.W. (2013). Catastrophic nuclear envelope collapse in cancer cell micronuclei. Cell.

[4] Sun L., Wu J., Du F., Chen X., Chen Z.J. (2013). Cyclic GMP-AMP synthase is a cytosolic DNA sensor that activates the type I interferon pathway. Science.

[5] Harding S.M., Benci J.L., Irianto J., Discher D.E., Minn A.J., Greenberg R.A. (2017). Mitotic progression following DNA damage enables pattern recognition within micronuclei. Nature.

[6] MacKenzie K.J., Carroll P., Martin C.A., Murina O., Fluteau A., Simpson D.J. (2017). CGAS surveillance of micronuclei links genome instability to innate immunity. Nature.

[7] Ablasser A., Goldeck M., Cavlar T., Deimling T., Witte G., Röhl I. (2013). cGAS produces a 2′-5′-linked cyclic dinucleotide second messenger that activates STING. Nature.

[8] Wu J., Sun L., Chen X., Du F., Shi H., Chen C. (2013). Cyclic GMP-AMP is an endogenous second messenger in innate immune signaling by cytosolic DNA. Science.

[9] Ishikawa H., Barber G.N. (2008). STING is an endoplasmic reticulum adaptor that facilitates innate immune signalling. Nature.

[10] Zhong B., Yang Y., Li S., Wang Y.Y., Li Y., Diao F. (2008). The adaptor protein MITA links virus-sensing receptors to IRF3 transcription factor activation. Immunity.

[11] Ablasser A., Chen Z.J. (2019). CGAS in action: expanding roles in immunity and inflammation. Science.

[12] Tanaka Y., Chen Z.J. (2012). STING specifies IRF3 phosphorylation by TBK1 in the cytosolic DNA signaling pathway. Sci. Signal..

[13] Fenton S.E., Saleiro D., Platanias L.C. (2021). Type i and ii interferons in the anti-tumor immune response. Cancers (Basel).

[14] Yang C.A., Huang H.Y., Chang Y.S., Lin C.L., Lai I.L., Chang J.G. (2017). DNA-sensing and nuclease gene expressions as markers for colorectal cancer progression. Oncol.

[15] Xia T., Konno H., Ahn J., Barber G.N. (2016). Deregulation of STING signaling in colorectal carcinoma constrains DNA damage responses and correlates with tumorigenesis. Cell Rep..

[16] Xia T., Konno H., Barber G.N. (2016). Recurrent loss of STING signaling in melanoma correlates with susceptibility to viral oncolysis. Cancer Res..

[17] Song S., Peng P., Tang Z., Zhao J., Wu W., Li H. (2017). Decreased expression of STING predicts poor prognosis in patients with gastric cancer. Sci. Rep..

[18] Mekers V.E., Kho V.M., Ansems M., Adema G.J. (2022). cGAS/cGAMP/STING signal propagation in the tumor microenvironment: key role for myeloid cells in antitumor immunity. Radiother. Oncol..

[19] Marcus A., Mao A.J., Lensink-Vasan M., Wang L.A., Vance R.E., Raulet D.H. (2018). Tumor-derived cGAMP triggers a STING-mediated interferon response in non-tumor cells to activate the NK cell response. Immunity.

[20] Schadt L., Sparano C., Schweiger N.A., Silina K., Cecconi V., Lucchiari G. (2019). Cancer-cell-intrinsic cGAS expression mediates tumor immunogenicity. Cell Rep..

[21] Carozza J.A., Böhnert V., Nguyen K.C., Skariah G., Shaw K.E., Brown J.A. (2020). Extracellular cGAMP is a cancer cell-produced immunotransmitter involved in radiation-induced anti-cancer immunity. Nat. Cancer.

[22] Zhou Y., Fei M., Zhang G., Liang W.C., Lin W.Y., Wu Y. (2020). Blockade of the phagocytic receptor MerTK on tumor-associated macrophages enhances P2X7R-Dependent STING activation by tumor-derived cGAMP. Immunity.

[23] Lu L., Yang C., Zhou X., Wu L., Hong X., Li W. (2023). STING signaling promotes NK cell antitumor immunity and maintains a reservoir of TCF-1+ NK cells. Cell Rep..

[24] Zhou C., Chen X., Planells-Cases R., Chu J., Wang L., Cao L. (2020). Transfer of cGAMP into bystander cells via LRRC8 volume-regulated anion channels augments STING-mediated interferon responses and anti-viral immunity. Immunity.

[25] Lahey L.J., Mardjuki R.E., Wen X., Hess G.T., Ritchie C., Carozza J.A. (2020). LRRC8A:C/E heteromeric channels are ubiquitous transporters of cGAMP. Mol. Cell.

[26] Ritchie C., Cordova A.F., Hess G.T., Bassik M.C., Li L. (2019). SLC19A1 is an importer of the immunotransmitter cGAMP. Mol. Cell.

[27] Luteijn R.D., Zaver S.A., Gowen B.G., Wyman S.K., Garelis N.E., Onia L. (2019). SLC19A1 transports immunoreactive cyclic dinucleotides. Nature.

[28] Cordova A.F., Ritchie C., Böhnert V., Li L. (2021). Human SLC46A2 is the dominant cGAMP importer in extracellular cGAMP-Sensing macrophages and monocytes. ACS Cent. Sci..

[29] Maltbaek J.H., Cambier S., Snyder J.M., Stetson D.B., Maltbaek J.H., Cambier S. (2022). Article ABCC1 transporter exports the immunostimulatory cyclic dinucleotide cGAMP article ABCC1 transporter exports the immunostimulatory cyclic dinucleotide cGAMP. Immunity.

[30] Zhang Z., Gao J., Liao X., Zhang Z., Cao X., Gong Y. (2025). ABCC10-mediated cGAMP efflux drives cancer cell radiotherapy resistance. Cell Death Differ.

[31] Ablasser A., Schmid-Burgk J.L., Hemmerling I., Horvath G.L., Schmidt T., Latz E. (2013). Cell intrinsic immunity spreads to bystander cells via the intercellular transfer of cGAMP. Nature.

[32] Ahn J., Xia T., Rabasa Capote A., Betancourt D., Barber G.N. (2018). Extrinsic phagocyte-dependent STING signaling dictates the immunogenicity of dying cells. Cancer Cell.

[33] Jentsch T.J. (2016). VRACs and other ion channels and transporters in the regulation of cell volume and beyond. Nat. Rev. Mol. Cell Biol.

[34] Voss F.K., Ullrich F., Munch J., Lazarow K., Lutte D., Mah N. (2014). Identification of LRRC8 heteromers as an essential component of the volume-regulated anion channel VRAC. Science.

[35] Qiu Z., Dubin A.E., Mathur J., Tu B., Reddy K., Miraglia L.J. (2014). SWELL1, a plasma membrane protein, is an essential component of volume-regulated anion channel. Cell.

[36] Deneka D., Sawicka M., Lam A.K.M., Paulino C., Dutzler R. (2018). Structure of a volume-regulated anion channel of the LRRC8 family. Nature.

[37] Kasuya G., Nakane T., Yokoyama T., Jia Y., Inoue M., Watanabe K. (2018). Cryo-EM structures of the human volume-regulated anion channel LRRC8. Nat. Struct. Mol. Biol..

[38] Kefauver J.M., Saotome K., Dubin A.E., Pallesen J., Cottrell C.A., Cahalan S.M. (2018). Structure of the human volume regulated anion channel. Elife.

[39] Planells-Cases R., Lutter D., Guyader C., Gerhards N.M., Ullrich F., Elger D.A. (2015). Subunit composition of VRAC channels determines substrate specificity and cellular resistance to P t-based anti-cancer drugs. EMBO J..

[40] Lutter D., Ullrich F., Lueck J.C., Kempa S., Jentsch T.J. (2017). Selective transport of neurotransmitters and modulators by distinct volume-regulated LRRC8 anion channels. J. Cell Sci..

[41] Concepcion A.R., Wagner L.E., Zhu J., Tao A.Y., Yang J., Khodadadi-Jamayran A. (2022). The volume-regulated anion channel LRRC8C suppresses T cell function by regulating cyclic dinucleotide transport and STING–p53 signaling. Nat. Immunol..

[42] Lu P., Ding Q., Li X., Ji X., Li L., Fan Y. (2019). SWELL1 promotes cell growth and metastasis of hepatocellular carcinoma in vitro and in vivo. EBioMedicine.

[43] Konishi T., Shiozaki A., Kosuga T., Kudou M., Shoda K., Arita T. (2019). LRRC8A expression influences growth of esophageal squamous cell carcinoma. Am. J. Pathol..

[44] Kurashima K., Shiozaki A., Kudou M., Shimizu H., Arita T., Kosuga T. (2021). LRRC8A influences the growth of gastric cancer cells via the p53 signaling pathway. Gastric Cancer.

[45] Cao L., Wang L., Li Z., Wei X., Ding J., Zhou C. (2025). Radiotherapy enhances anticancer CD8 T cell responses by cGAMP transfer through LRRC8A/C regulated anion channels. Sci Immunol.

[46] Demaria O., De Gassart A., Coso S., Gestermann N., Di Domizio J., Flatz L. (2015). STING activation of tumor endothelial cells initiates spontaneous and therapeutic antitumor immunity. Proc. Natl. Acad. Sci. U. S. A..

[47] Lau J., Cheung J., Navarro A., Lianoglou S., Haley B., Totpal K. (2017). Tumour and host cell PD-L1 is required to mediate suppression of anti-tumour immunity in mice. Nat. Commun..

[48] Shields N.J., Peyroux E.M., Ferguson A.L., Steain M., Neumann S., Young S.L. (2023). Late-stage MC38 tumours recapitulate features of human colorectal cancer – implications for appropriate timepoint selection in preclinical studies. Front. Immunol..

[49] Caisová V., Vieru A., Kumžáková Z., Glaserová S., Husníková H., Vácová N. (2016). Innate immunity based cancer immunotherapy: B16-F10 murine melanoma model. BMC Cancer.

[50] Zhang Z., Zhou H., Ouyang X., Dong Y., Sarapultsev A., Luo S. (2022). Multifaceted functions of STING in human health and disease: from molecular mechanism to targeted strategy. Signal Transduct. Target. Ther..

[51] Chen X., Wang L., Cao L., Li T., Li Z., Sun Y. (2021). Regulation of anion channel LRRC8 volume-regulated anion channels in transport of 2′3′-Cyclic GMP–AMP and Cisplatin under steady state and inflammation. J. Immunol..

[52] Tomayko M.M., Reynolds C.P. (1989). Determination of subcutaneous tumor size in athymic (nude) mice. Cancer Chemother. Pharmacol..

[53] Kim J.C., Liu X., Fitzgerald K., Eng J.S., Orf J., O’Brien S.A. (2023). Brief report: STING expressed in tumor and non-tumor compartments has distinct roles in regulating anti-tumor immunity. Cancer Immunol. Immunother..

[54] Wang L., Cao L., Li Z., Shao Z., Chen X., Huang Z. (2024). ATP-elicited cation fluxes promote volume-regulated anion channel LRRC8/VRAC transport cGAMP for antitumor immunity. J. Immunol..

[55] Ahn J., Gutman D., Saijo S., Barber G.N. (2012). STING manifests self DNA-dependent inflammatory disease. Proc. Natl. Acad. Sci. U. S. A..

[56] de Vieira R.S., Nascimento M.S., Noronha I.H., Vasconcelos J.R.C., Benvenuti L.A., Barber G.N. (2022). STING signaling drives production of innate cytokines, generation of CD8+ T cells and enhanced protection against Trypanosoma cruzi infection. Front. Immunol..

[57] Ren H., Zhang J., Jiang Y., Hao S., You J., Yin Z. (2024). C-di-GMP@ZIF-8 nanocomposite injectable hydrogel based on modified chitosan and hyaluronic acid for infected wound healing by activating STING signaling. Int. J. Biol. Macromol.

[58] Woo S.R., Fuertes M.B., Corrales L., Spranger S., Furdyna M.J., Leung M.Y.K. (2014). STING-dependent cytosolic DNA sensing mediates innate immune recognition of immunogenic tumors. Immunity.

[59] Corrales L., Glickman L.H., McWhirter S.M., Kanne D.B., Sivick K.E., Katibah G.E. (2015). Direct activation of STING in the tumor microenvironment leads to potent and systemic tumor regression and immunity. Cell Rep..

[60] Glück S., Guey B., Gulen M.F., Wolter K., Kang T.W., Schmacke N.A. (2017). Innate immune sensing of cytosolic chromatin fragments through cGAS promotes senescence. Nat. Cell Biol..

[61] Kumar L., Chou J., Yee C.S.K., Borzutzky A., Vollmann E.H., von Andrian U.H. (2014). Leucine-rich repeat containing 8A (LRRC8A) is essential for T lymphocyte development and function. J. Exp. Med..

[62] Lopez-Cayuqueo K.I., Planells-Cases R., Pietzke M., Oliveras A., Kempa S., Bachmann S. (2022). Renal deletion of LRRC8/VRAC channels induces proximal tubulopathy. J. Am. Soc. Nephrol..

[63] Lv H., Zong Q., Chen C., Lv G., Xiang W., Xing F. (2024). TET2-mediated tumor cGAS triggers endothelial STING activation to regulate vasculature remodeling and anti-tumor immunity in liver cancer. Nat. Commun..

[64] Stuhlmann T., Planells-Cases R., Jentsch T.J. (2018). LRRC8/VRAC anion channels enhance β-cell glucose sensing and insulin secretion. Nat. Commun..

[65] Stranges P.B., Watson J., Cooper C.J., Choisy-Rossi C.M., Stonebraker A.C.C., Beighton R.A. (2007). Elimination of antigen-presenting cells and autoreactive T cells by fas contributes to prevention of autoimmunity. Immunity.

[66] Li T., Cheng H., Yuan H., Xu Q., Shu C., Zhang Y. (2016). Antitumor activity of cGAMP via stimulation of cGAS-cGAMP-STING-IRF3 mediated innate immune response. Sci. Rep..

[67] Hoffmann E.K., Lambert I.H., Pedersen S.F. (2009). Physiology of cell volume regulation in vertebrates. Physiol. Rev..

[68] Hoffmann E.K., Sørensen B.H., Sauter D.P.R., Lambert I.H. (2015). Role of volume-regulated and calcium-activated anion channels in cell volume homeostasis, cancer and drug resistance. Channels.

[69] Carpanese V., Festa M., Prosdocimi E., Bachmann M., Sadeghi S., Bertelli S. (2024). Interactomic exploration of LRRC8A in volume-regulated anion channels. Cell Death Discov..

[70] Liu T., Stauber T. (2019). The volume-regulated anion channel lrrc8/vrac is dispensable for cell proliferation and migration. Int. J. Mol. Sci..

[71] Zhang Y., Li Y., Thompson K.N., Stoletov K., Yuan Q., Bera K. (2022). Polarized NHE1 and SWELL1 regulate migration direction, efficiency and metastasis. Nat. Commun..

[72] Lewicky J.D., Martel A.L., Gupta M.R., Roy R., Rodriguez G.M., Vanderhyden B.C. (2023). Conventional DNA-damaging cancer therapies and emerging cGAS-STING activation: a review and perspectives regarding immunotherapeutic potential. Cancers (Basel).

[73] Tishchenko A., Romero N., van Waesberghe C., Delva J.L., Vickman O., Smith G.A. (2025). Pseudorabies virus infection triggers pUL46-mediated phosphorylation of connexin-43 and closure of gap junctions to promote intercellular virus spread. PLoS Pathog..

[74] Sivick K.E., Desbien A.L., Glickman L.H., Reiner G.L., Corrales L., Surh N.H. (2018). Magnitude of therapeutic STING activation determines CD8+ T cell-mediated anti-tumor immunity. Cell Rep..

[75] Jneid B., Bochnakian A., Hoffmann C., Delisle F., Djacoto E., Sirven P. (2025). Selective STING stimulation in dendritic cells primes antitumor T cell responses. Sci. Immunol..

[76] Shae D., Becker K.W., Christov P., Yun D.S., Lytton-Jean A.K.R., Sevimli S. (2019). Endosomolytic polymersomes increase the activity of cyclic dinucleotide STING agonists to enhance cancer immunotherapy. Nat. Nanotechnol..

[77] Cheng N., Watkins-Schulz R., Junkins R.D., David C.N., Johnson B.M., Montgomery S.A. (2018). A nanoparticle-incorporated STING activator enhances antitumor immunity in PD-L1-insensitive models of triple-negative breast cancer. JCI Insight.

[78] Liu Y., Crowe W.N., Wang L., Lu Y., Petty W.J., Habib A.A. (2019). An inhalable nanoparticulate STING agonist synergizes with radiotherapy to confer long-term control of lung metastases. Nat. Commun..

[79] Wu J., Dobbs N., Yang K., Yan N. (2020). Interferon-independent activities of mammalian STING mediate antiviral response and tumor immune evasion. Immunity.

[80] Sauer J.D., Sotelo-Troha K., Von Moltke J., Monroe K.M., Rae C.S., Brubaker S.W. (2011). The N-ethyl-N-nitrosourea-induced Goldenticket mouse mutant reveals an essential function of sting in the in vivo interferon response to Listeria monocytogenes and cyclic dinucleotides. Infect. Immun..

