# Supplementary material for: A protective cGAMP-mediated anti-tumor immune response can proceed without LRRC8/VRAC channels
Source: J Biol Chem. 2025 Dec 17;302(2):111060. doi: 10.1016/j.jbc.2025.111060 (PMC12828768; doi:10.1016/j.jbc.2025.111060)
Supplement: Supplementary Material [file mmc1.pdf]

Supporting Information

A protective cGAMP-mediated anti-tumor immune response can proceed without LRRC8/VRAC channels

Fabian M. B. Thöne, Maya M. Polovitskaya Uta E. Höpken, Armin Rehm, Thomas J. Jentsch

Supporting informaition containes:

- Supporting Figure 1 (Figure S1)
- Supporting Figure 2 (Figure S2)
- Supporting Figure 3 (Figure S3)
- Supporting Figure 4 (Figure S4)
- Supporting Figure 5 (Figure S5)
- Supporting Figure 6 (Figure S6)
- Supporting Figure 7 (Figure S7)
- Supporting Figure 8 (Figure S8)
- Supporting Figure 9 (Figure S9)
- Supporting Figure 10 (Figure S10)
- Supporting Figure 11 (Figure S11)
- Supporting Figure 12 (Figure S12)
- Supporting Table 1 (Table S1)
- Supporting Table 2 (Table S2)

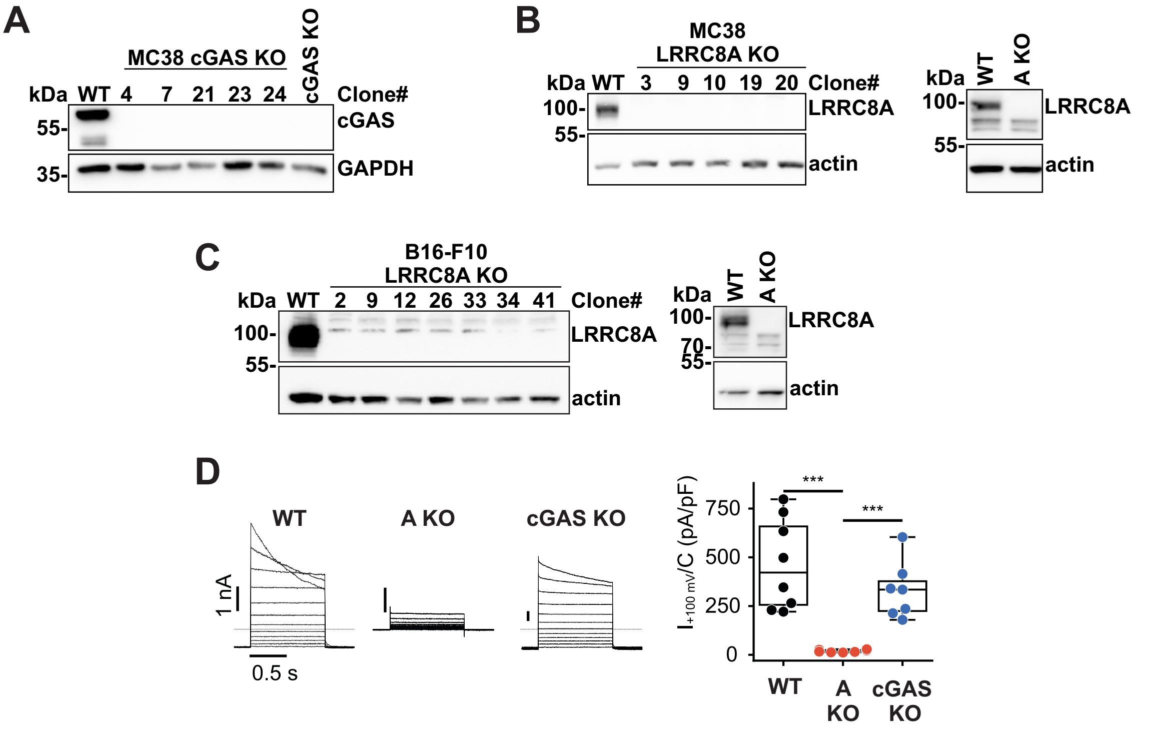

Figure S1. *A*–*C*, confirmation of cGAS (*A*) and LRRC8A (*B* & *C*) knockout in single clones and polyclonal (cGAS KO or A KO) MC38 (*A* & *B*) and B16-F10 (*C*) cell lines. Polyclonal cell lines were generated by combining equal proportions of all depicted monoclonal cell lines. *D*, whole-cell patch-clamp recordings of hypotonicity-induced currents in MC38 cells (n = 7–8). Example traces were obtained from cells stimulated by 25% reduction of extracellular osmolarity at voltages ranging from −100 to +120 mV. Note the variable time-dependent inactivation at inside-positive potentials in WT and cGAS-deficient cells. Steady-state current densities were obtained at +100 mV after at least 5 minutes of hypotonic stimulation. Data are represented as mean ± SD. Normality was confirmed (Shapiro-Wilk test) and data was analyzed using unpaired Welch´s *t*-test. False discovery rate was controlled using the Benjamini-Hochberg procedure. *$p < 0.05$; **$p < 0.01$; ***$p < 0.001$.

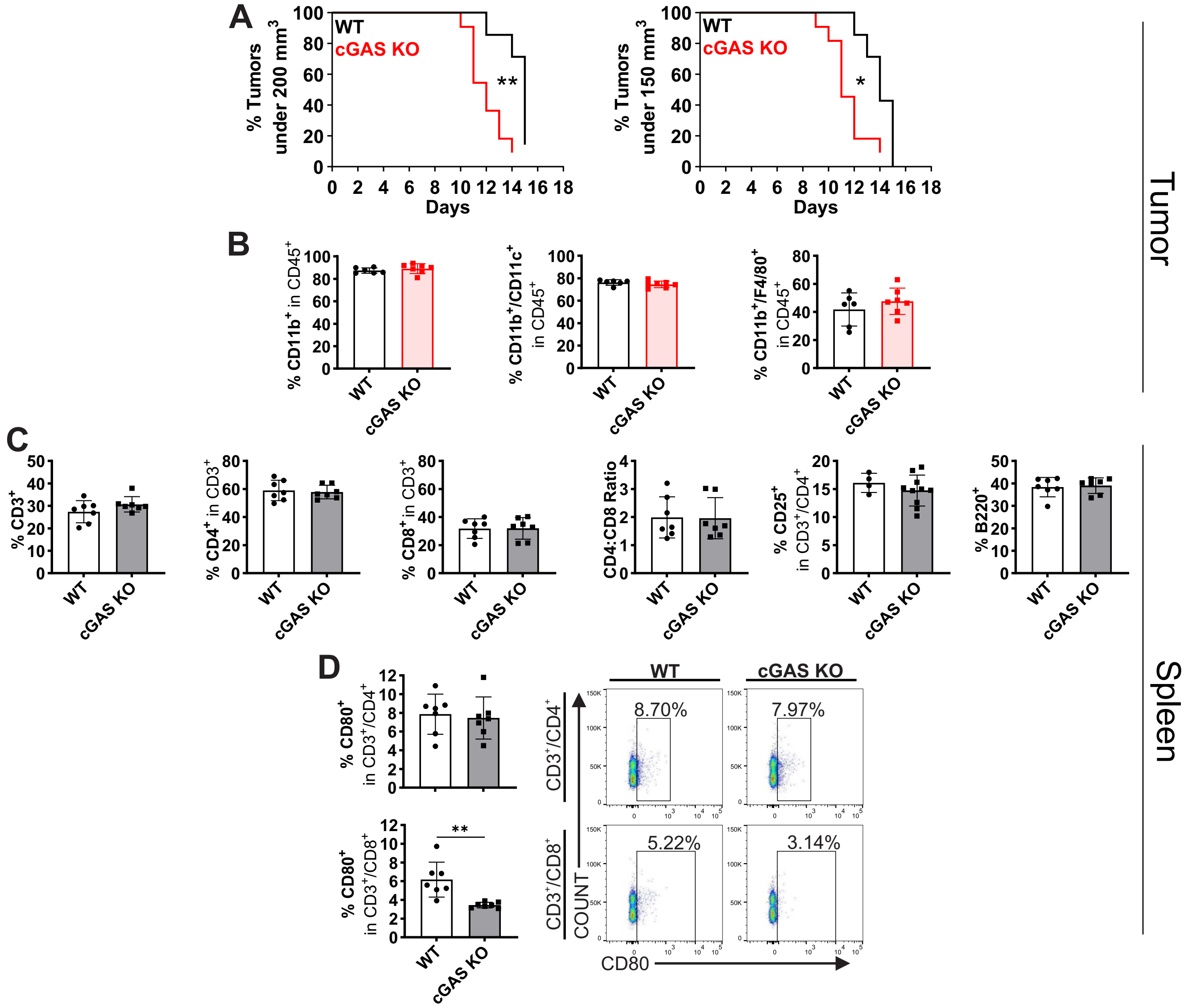

Figure S2. *A*, WT (*n* = 7) or cGAS-deficient (*n* = 11) MC38 cells were subcutaneously injected into recipient WT mice. Tumor volumes were plotted as Kaplan-Meier curves using volumes of 150 mm^3 or 200 mm^3 as cutoffs. *p < 0.05; **p < 0.01 (log-rank Mantel-Cox test). *B*, flow cytometric analysis of myeloid cell populations in WT (*n* = 6) or cGAS-deficient (*n* = 7) MC38 tumors. *C*, flow cytometric analysis of splenic T and B cells in mice bearing WT or cGAS-deficient tumors (*n* = 7). *D*, flow cytometric quantification of CD80-positive T cells in spleens of mice bearing WT or cGAS-deficient tumors (*n* = 7), including representative flow cytometry plots. Data in panels *B*–*D* are represented as mean ± SD. Normality was confirmed for all data in panel *D* (Shapiro-Wilk test) and Welch´s *t*-test was used for analysis. **p < 0.01. Note that the control group (WT MC38 tumors in WT mice) was partially shared with experiments involving *Lrrc8c*$^{-/-}$ mice; therefore, portions of the data presented here are also included in figures 6 and S5.

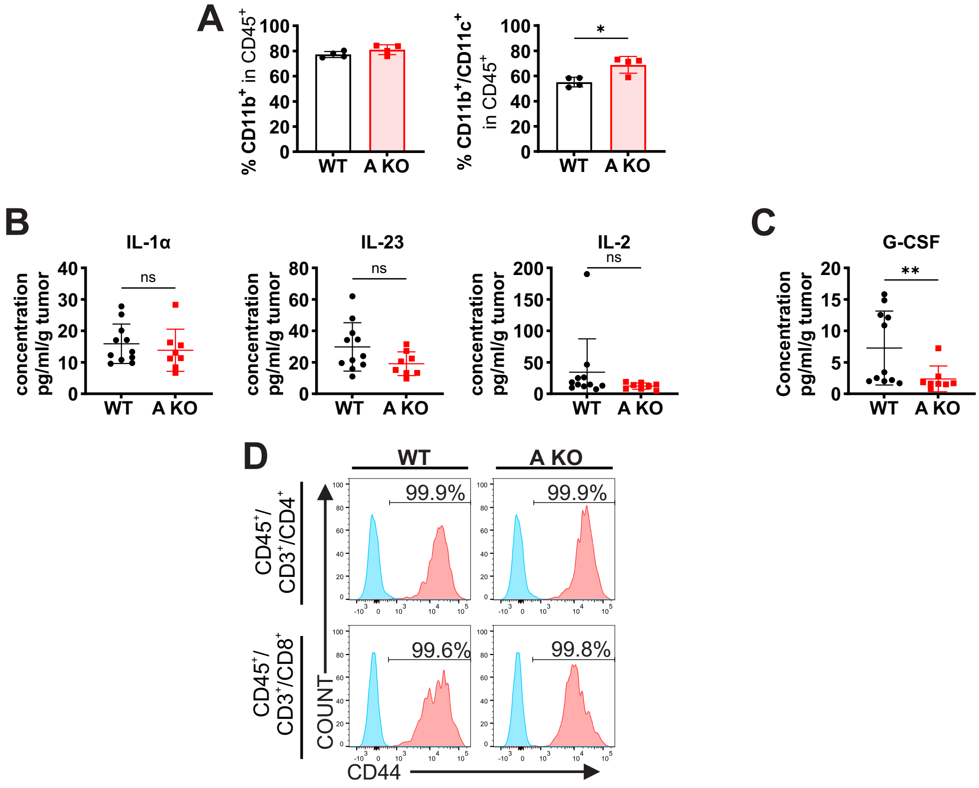

Figure S3. *A*, flow cytometric analysis of myeloid cell populations in WT and LRRC8A-deficient MC38 tumors ($n = 4$). *B* and *C*, blood serum cytokine concentrations in mice bearing WT ($n = 11$) or LRRC8A-deficient ($n = 8$) MC38 tumors. Blood serum was obtained at the time of tumor resection and subjected to multiplex cytokine assay. Serum cytokine concentrations from individual mice were normalized to the weight of the corresponding tumor. *D*, representative flow cytometry plots of the percentage of CD44-positive T cells (red) in WT and LRRC8A-deficient MC38 tumors. Isotype control staining (blue) was used to define the CD44-positive gate. Data are represented as mean ± SD. All data in panels *A*–*C* were tested for normality (Shapiro-Wilk test). Normally distributed data (CD11b, CD11b/CD11c, IL-1α and IL-23) were analyzed using unpaired Welch´s *t*-test, while Mann-Whitney U test was used for non-normally distributed data (IL-2 and G-CSF). $*p < 0.05$; $**p < 0.01$.

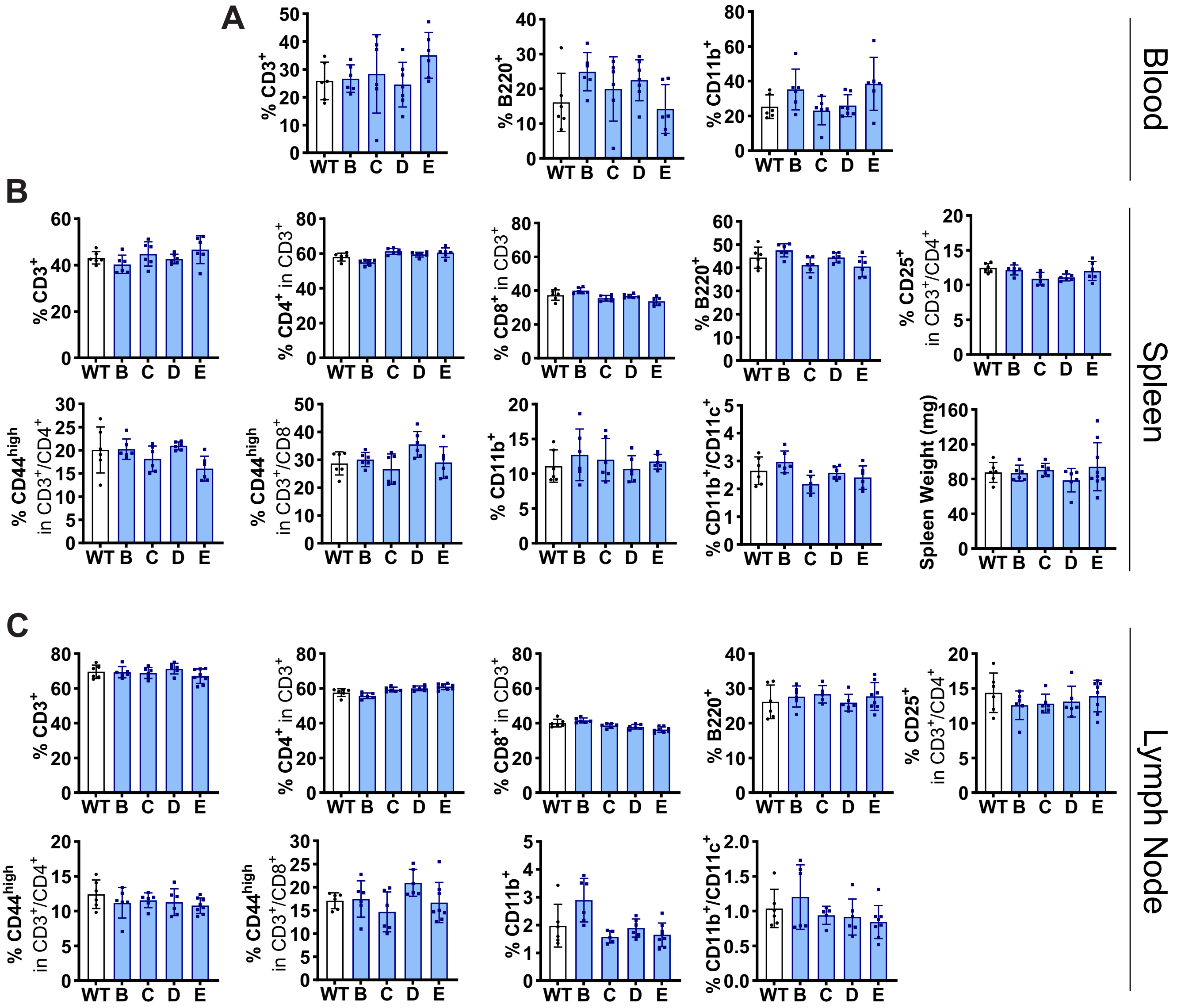

Figure S4. *A*–*C*, flow cytometric analysis of immune cells in peripheral blood (*A*), spleens (*B*) and lymph nodes (*C*) of *Lrrc8b*$^{-/-}$, -*c*$^{-/-}$, -*d*$^{-/-}$ or -*e*$^{-/-}$ mice under homeostatic conditions, as well as weights of spleens (*B*). Peripheral blood, spleens and lymph nodes (inguinal and mesenteric) were extracted from 8 to 12 weeks old mice (*n* = 6–7; at least 3 males and 3 females) and subjected to flow cytometry. Data are represented as mean ± SD.

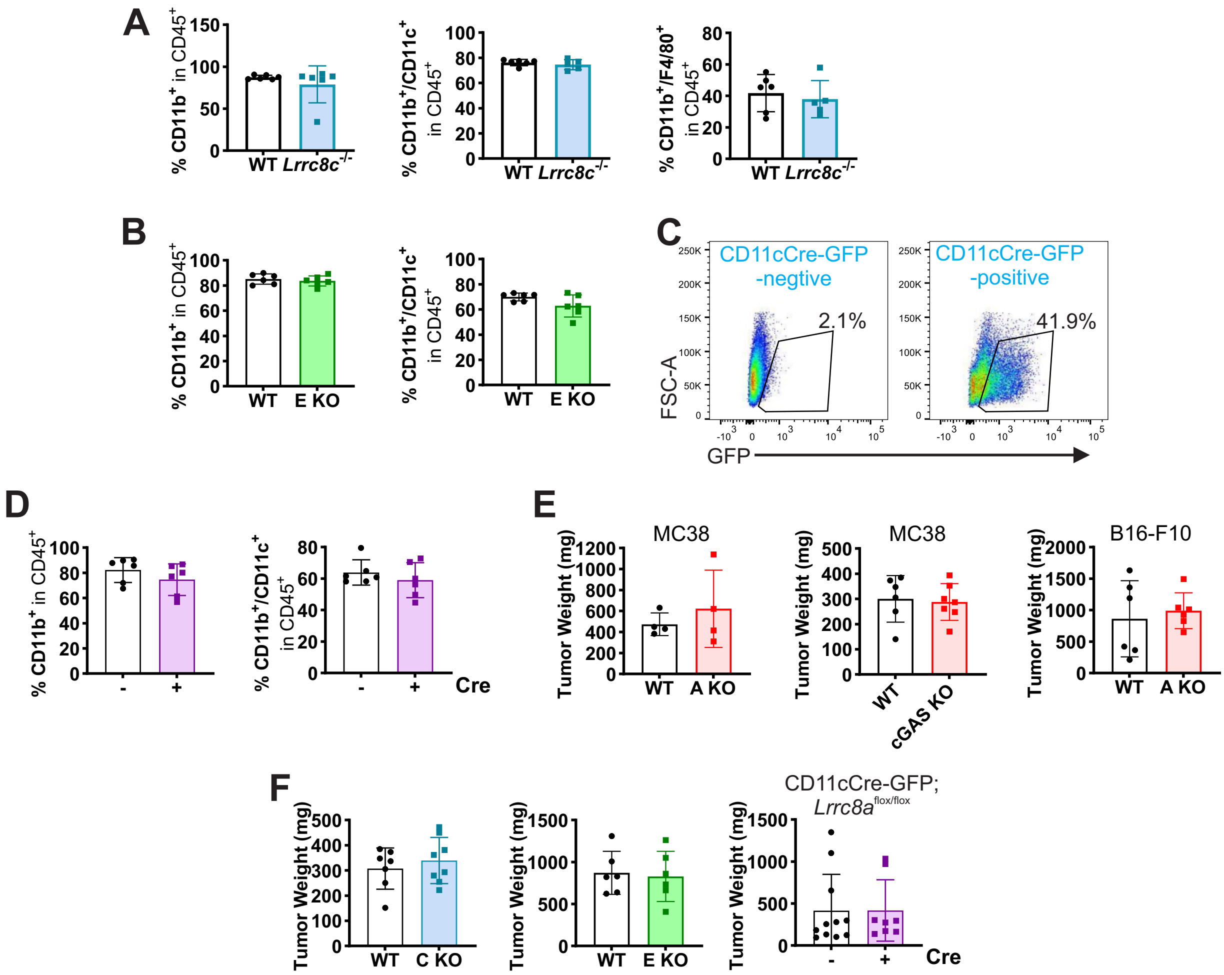

Figure S5. *A* and *B*, flow cytometric analysis of myeloid cell populations in MC38 tumors from *Lrrc8c*$^{-/-}$ (C KO; *n* = 5–6) (*A*) and *Lrrc8e*$^{-/-}$ (E KO; *n* = 6) (*B*) mice. *C*, flow cytometric quantification of GFP-positive cells within the CD45-positive population in MC38 tumors from CD11cCre-GFP;*Lrrc8a*$^{flox/flox}$ mice. *D*, flow cytometric analysis of myeloid cell populations in MC38 tumors from CD11cCre-GFP;*Lrrc8a*$^{flox/flox}$ mice (*n* = 6). *E* and *F*, weights of tumors selected for flow cytometry analysis investigating LRRC8A (MC38: *n* = 4, B16-F10: *n* = 6) and cGAS KO (*n* = 6–7) tumors (E) or WT tumors in mice with *Lrrc8* deletions (*Lrrc8c*$^{-/-}$: n = 7–8, *Lrrc8e*$^{-/-}$: n = 6, CD11cCre-GFP$^{+/-}$;*Lrrc8a*$^{flox/flox}$: n = 8–11) (F). Data are represented as mean ± SD. Note that the control group for experiments with *Lrrc8c*$^{-/-}$ animals (WT MC38 tumors in WT mice) was partially shared with experiments involving cGAS KO tumors; therefore, portions of the data presented here are also included in figures 3 and S2. Flow cytometry plots shown in *C* are also included in Figure S9.

MC38 Tumors

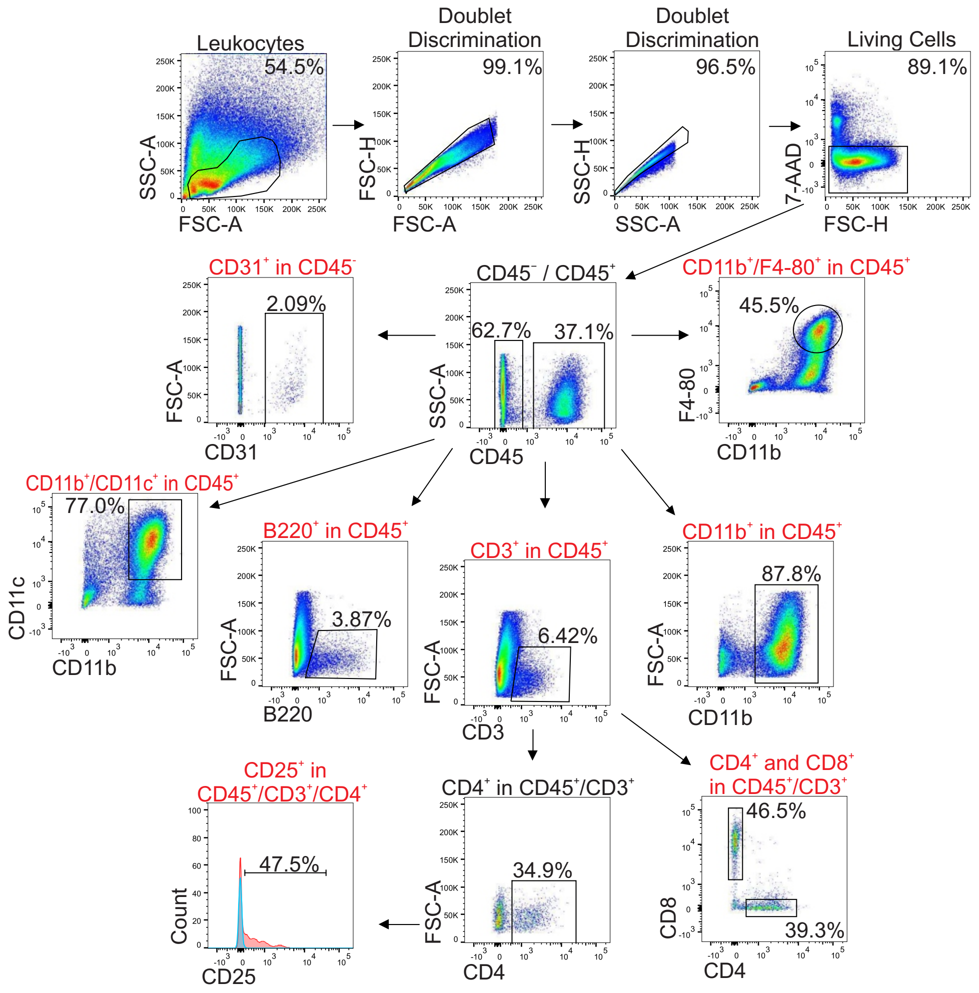

Figure S6. Flow cytometry gating strategy used to identify cell populations in MC38 tumors. Populations used for analysis are highlighted with red headings. Note that a different gating strategy was used for the analysis of T cells in MC38 tumors from CD11cCre-GFP;*Lrrc8a*$^{flox/flox}$ mice, shown in figure S9.

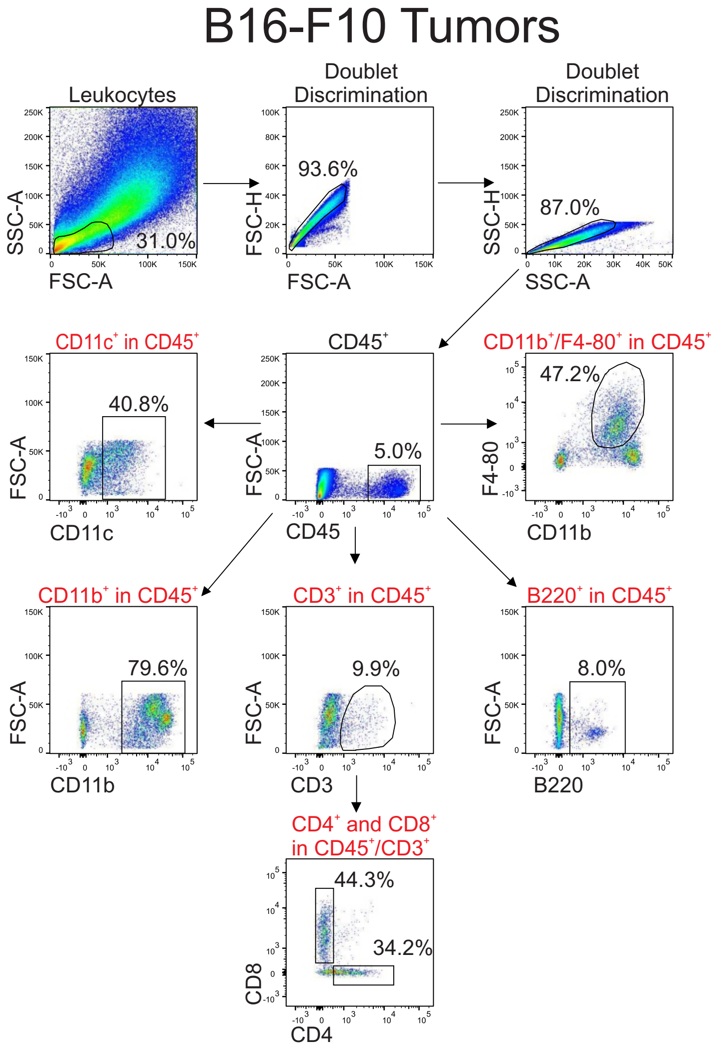

Figure S7. Flow cytometry gating strategy used to identify cell populations in B16-F10 tumors. Populations used for analysis are highlighted with red headings.

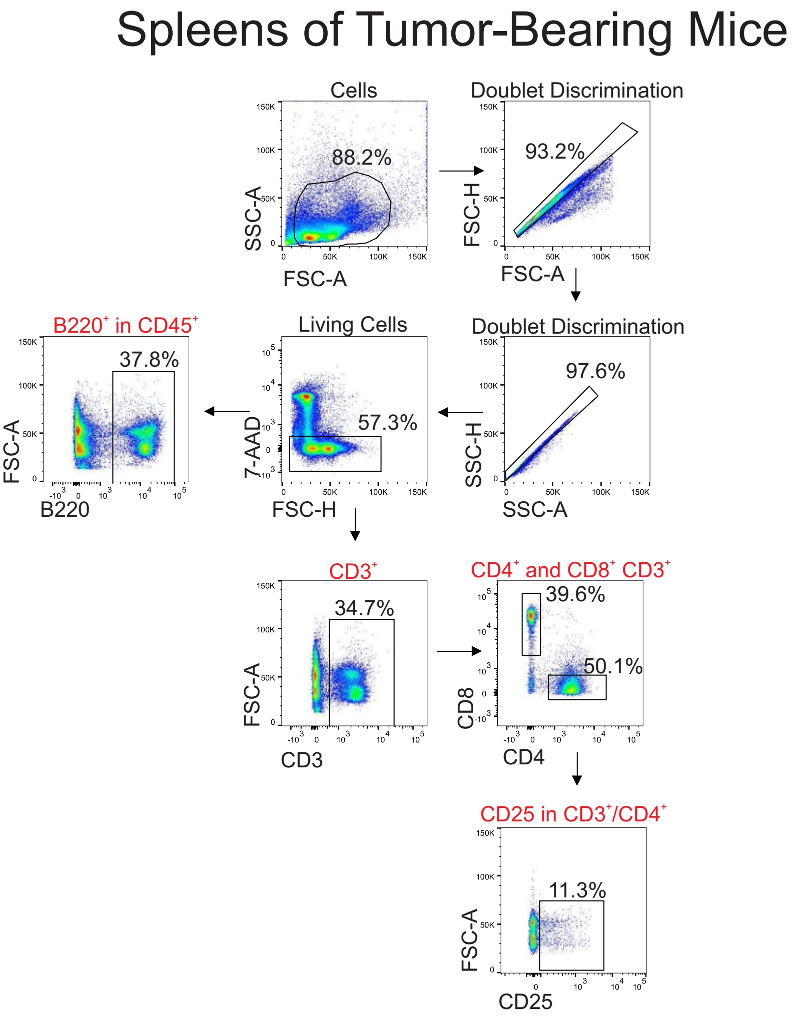

Figure S8. Flow cytometry gating strategy used to identify immune cell populations in spleens of MC38 tumor-bearing mice. Populations used for analysis are highlighted with red headings.

T Cell Gating in MC38 Tumors from CD11cCre-GFP;*Lrrc8a*$^{flox/flox}$ Mice

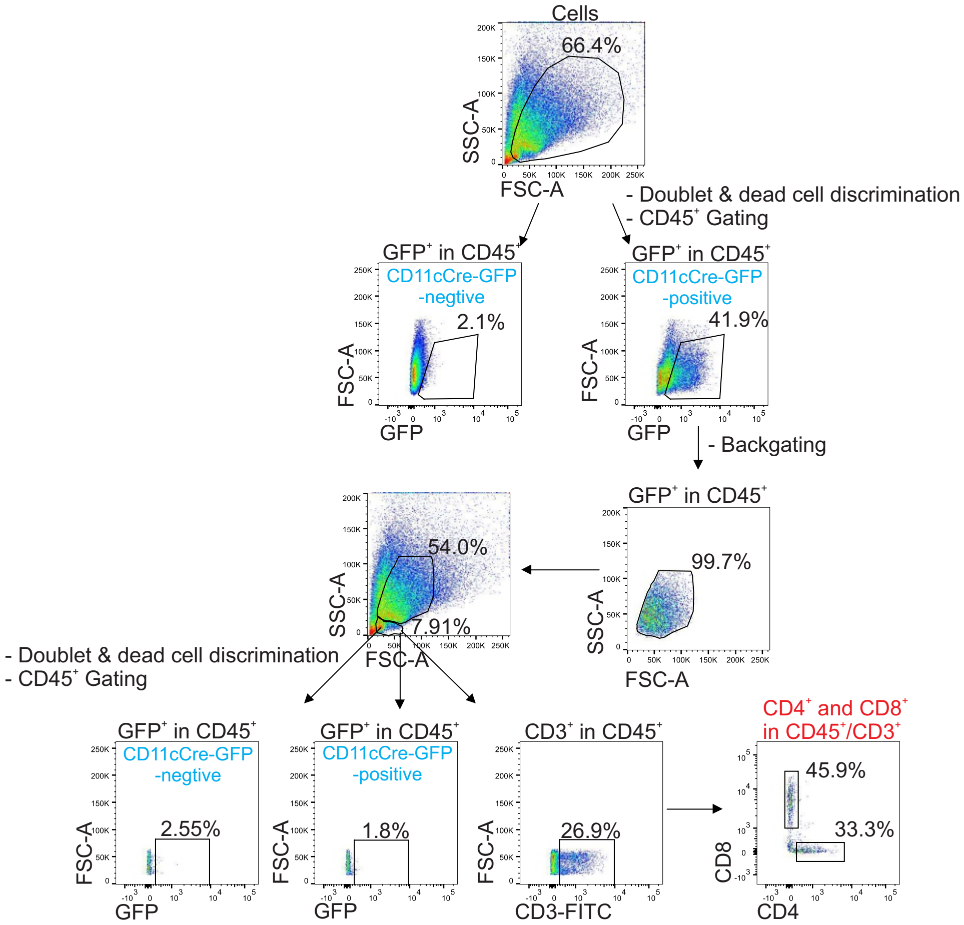

Figure S9. Flow cytometry gating strategy used to identify T cell populations in MC38 tumors from CD11cCre-GFP;*Lrrc8a*$^{flox/flox}$ mice. This special gating strategy was necessary due to spectral overlap of GFP with FITC-labeled anti-CD3 antibody. The GFP-positive population was identified in tumors from CD11cCre-GFP^{+} mice stained with an antibody mix lacking FITC-CD3 and located in the SSC-A vs. FSC-A blot through backgating. Subsequently, a stringent gate was applied excluding GFP-positive cells. Populations used for analysis are highlighted with red headings. Mouse genotypes are highlighted in blue. Note that gates showing GFP^{+} cells are also included in Figure S5C.

Blood

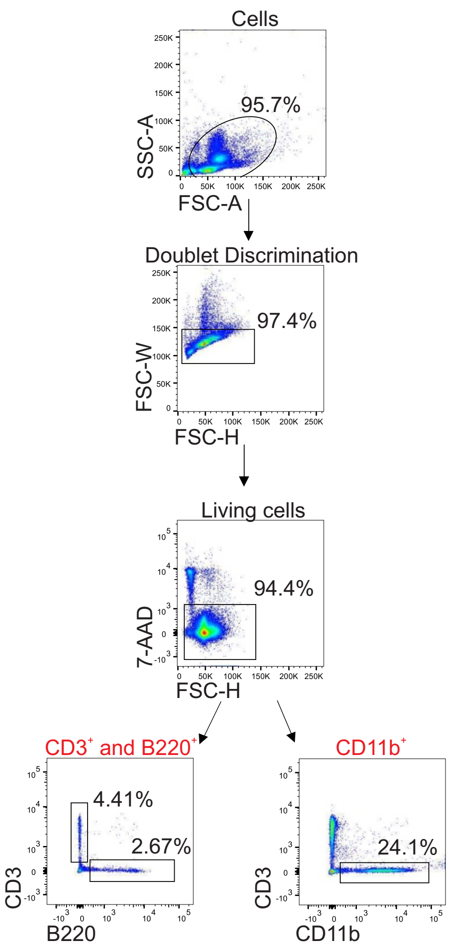

Figure S10. Flow cytometry gating strategy used to identify immune cell populations in peripheral blood of *Lrrc8b*$^{-/-}$, -*c*$^{-/-}$, -*d*$^{-/-}$ and -*e*$^{-/-}$ mice under homeostatic conditions. Populations used for analysis are highlighted with red headings.

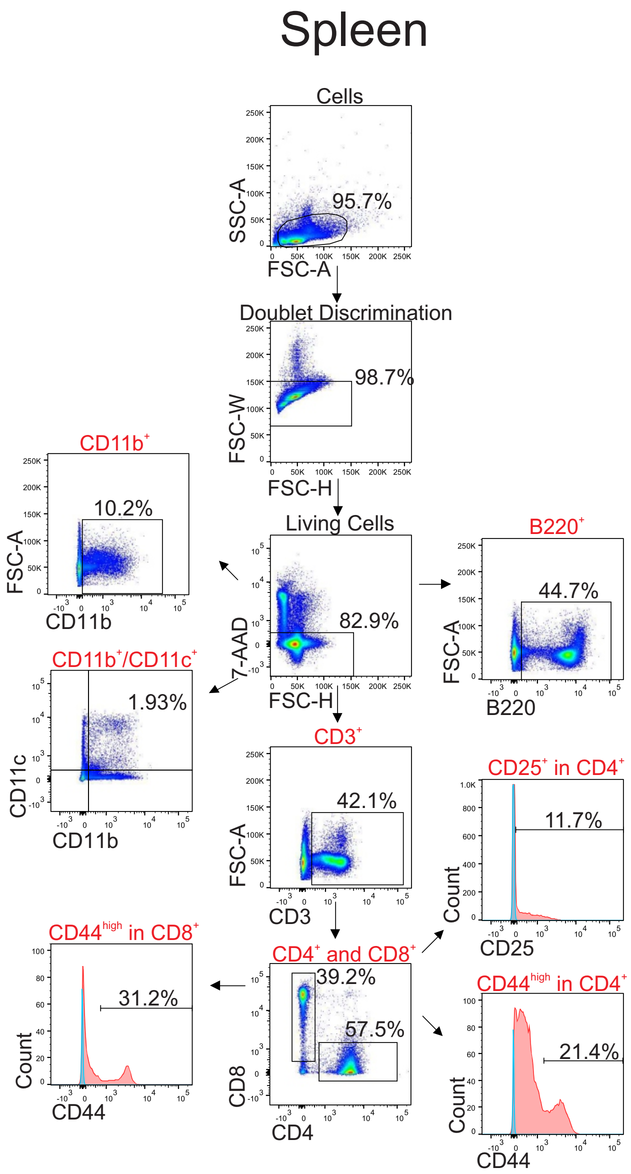

Figure S11. Flow cytometry gating strategy used to identify immune cell populations in spleens of *Lrrc8b*$^{-/-}$, -*c*$^{-/-}$, -*d*$^{-/-}$ and -*e*$^{-/-}$ mice under homeostatic conditions. Populations used for analysis are highlighted with red headings.

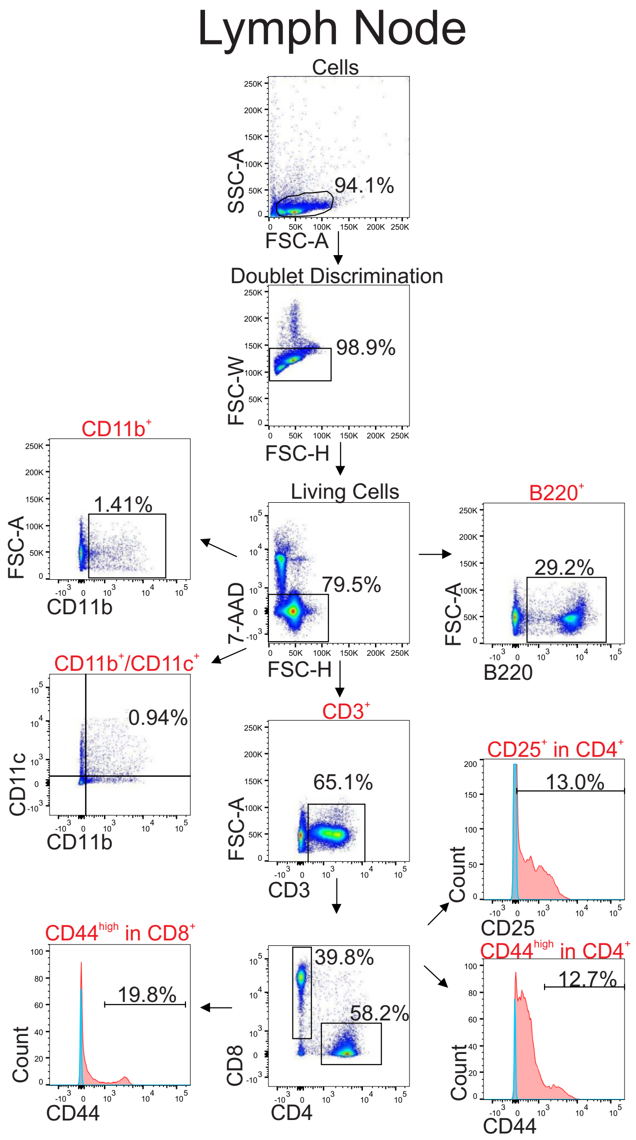

Figure S12. Flow cytometry gating strategy used to identify immune cell populations in lymph nodes (inguinal and mesenteric) of *Lrrc8b*$^{-/-}$, -*c*$^{-/-}$, -*d*$^{-/-}$ and -*e*$^{-/-}$ mice under homeostatic conditions. Populations used for analysis are highlighted with red headings.

Table S1: Oligonucleotides

Target gene	Sequence (5´ to 3´)	Purpose
Lrrc8a	CACCGTCGACACCAGTACAACTACG	CRISPR target sequence
Lrrc8a	AAACCGTAGTTGTACTGGTGTCGAC	CRISPR target sequence
cGAS	CACCGAAACGCAAAGATATCTCGG	CRISPR target sequence
cGAS	AAACCCGAGATATCTTTGCGTTTC	CRISPR target sequence
No Target	CACCGCACTACCAGAGCTAACTCA	CRISPR non-target control
No Target	AAACTGAGTTAGCTCTGGTAGTGC	CRISPR non-target control
Lrrc8a	CTTACCCCAACTCCACAGTC	Amplification of genomic locus (forward)
Lrrc8a	TGCTCCCCTTCCTTCTTGTC	Amplification of genomic locus (reverse)
cGAS	AAGTCGTAAGGGGACCTAGC	Amplification of genomic locus (forward)
cGAS	GTCTTGACGCTCACCTTCACA	Amplification of genomic locus (reverse)
Cxcl10	CTCATCCTGCTGGGTCTGAGT	qRT-PCR Primer (forward)
Cxcl10	CCTATGGCCCTCATTCTCACTG	qRT-PCR Primer (reverse)
Mx2	GTGCGGCCCTGCATTGACCT	qRT-PCR Primer (forward)
Mx2	GGCCACTCCAGACAGTGCTTCTAGT	qRT-PCR Primer (reverse)
Beta-actin	TGTGATGGTGGGAATGGGTCAGAA	qRT-PCR Primer (forward)
Beta-actin	TGTGGTGCCAGATCTTCTCCATGT	qRT-PCR Primer (reverse)
Lrrc8a-lox	CTGCAGAACCTCCAGAACCT	Mouse Genotyping (forward)
Lrrc8a-lox	TGTTGGGAGACAGATACCAC	Mouse Genotyping (reverse)
Lrrc8b WT	AGAAAGGGGAAATTCATTCAGCGGC	Mouse Genotyping (forward)
Lrrc8b WT	TCCCTTAGTTTGGGGACCAACTGGG	Mouse Genotyping (reverse)
Lrrc8b KO	GCTACCATTACCAGTTGGTCTGGTGTC	Mouse Genotyping (forward)
Lrrc8b KO	ACAAGATCTTCTCAAACAAAAGTCAAGC	Mouse Genotyping (reverse)
Lrrc8c WT	TTGTAACAAAGTGGAGAGCC	Mouse Genotyping (forward)
Lrrc8c WT	AGGAAAGGCATTAGGACTACC	Mouse Genotyping (reverse)
Lrrc8c KO	GGATCTCCTGTCATCTCACC	Mouse Genotyping (forward)
Lrrc8c KO	TGGTACATATGATGAGACTCA	Mouse Genotyping (reverse)
Lrrc8d	CCAACTTACCGAATCCTGA	Mouse Genotyping (forward)
Lrrc8d	TAGAGAATTCGAAACACTTTCCTAA	Mouse Genotyping (reverse)
Lrrc8e	AGTGGCGGAGTTCAAGCAGTTCA	Mouse Genotyping (forward)
Lrrc8e	GCCTTGCCACATCCCACTCATT	Mouse Genotyping (reverse)
CD11c *(Itax)* -Cre	ACTTGGCAGCTGTCTCCAAG	Mouse Genotyping (forward)
CD11c *(Itax)* -Cre	GCGAACATCTTCAGGTTCTG	Mouse Genotyping (reverse)

Table S2: Antibodies

Antibody	Application	Dilution	Source
Rabbit anti-LRRC8A	WB, primary antibody	1:1000	Jentsch Lab*
Rabbit anti-LRRC8B	WB, primary antibody	1:750	Jentsch Lab**
Rabbit anti-LRRC8C	WB, primary antibody	1:1000	Jentsch Lab***
Rabbit anti-LRRC8D	WB, primary antibody	1:1000	Jentsch Lab****
Rabbit anti-LRRC8E	WB, primary antibody	1:1000	Jentsch Lab*****
Rabbit anti-cGAS	WB, primary antibody	1:1000	Cell Signaling #83623
Rabbit anti-STING	WB, primary antibody	1:1000	Cell Signaling #13647
Rabbit anti-TBK1	WB, primary antibody	1:1000	Cell Signaling #3504
Rabbit anti-p-TBK1	WB, primary antibody	1:1000	Cell Signaling #5483
Rabbit anti-IRF3	WB, primary antibody	1:1000	Cell Signaling #4302
Rabbit anti-p-IRF3	WB, primary antibody	1:1000	Cell Signaling #4947
Mouse anti-beta-ACTIN	WB, primary antibody	1:10000	Sigma-Aldrich #A2228
Rabbit anti-GAPDH	WB, primary antibody	1:2000	Sigma-Aldrich #G8795
Goat anti-Rabbit	WB, second. antibody	1:10000	Cell Signaling #35401
Goat anti-Mouse	WB, second. antibody	1:10000	Cell Signaling #7076
PE/Cyanine7 anti-mouse CD45	Flow Cytometry	1:600	Biolegend 103114
FITC anti-mouse CD3	Flow Cytometry	1:300	Biolegend 100306
Pacific Blue™ anti-mouse CD4	Flow Cytometry	1:300	Biolegend 100428
APC anti-mouse CD8a	Flow Cytometry	1:300	Biolegend 100712
PE anti-mouse CD25	Flow Cytometry	1:100	Biolegend 102008
PE anti-mouse CD44	Flow Cytometry	1:200	Biolegend 103008
PE anti-mouse CD11b	Flow Cytometry	1:600	Biolegend 101208
APC anti-mouse CD11b	Flow Cytometry	1:400	Biolegend 101212
FITC anti-mouse CD11c	Flow Cytometry	1:100	Biolegend 117306
APC anti-mouse B220	Flow Cytometry	1:400	Biolegend 103212
PE anti-mouse B220	Flow Cytometry	1:600	Biolegend 103208
PE anti-mouse CD31	Flow Cytometry	1:600	Biolegend 160204
Anti-mouse CD16/32	Flow Cytometry	1:100	Biolegend 101302
FITC Armenian Hamster IgG Isotype CTR	Flow Cytometry	1:300	Biolegend 400906
Pacific Blue™ Rat IgG2b, $_\kappa$ Isotype Ctrl	Flow Cytometry	1:300	Biolegend 400627
APC Rat IgG2a, $_\kappa$ Isotype Ctrl	Flow Cytometry	1:300	Biolegend 400512
PE Rat IgG2b, K Isotype Ctrl	Flow Cytometry	1:200	Biolegend 400608
Rat anti-CD31	Immunofluorescence	1:500	BD Bioscience 553369
Alexa-647 Donkey anti-Rat	Immunofluorescence	1:10000	Jackson 712-605-153

* target peptide: QRTKSRIEQGIVDRSE, Database number: #1339 (Voss et al., *Science 2014*)
** target peptide: QSLPYPQPGLESPGIESPT, Database number: #1346 (Stuhlmann et al. *Nat. Commun 2018*)
*** target peptide: EDALFETLPSDVREQMKAD, Database number: #1347 R62/D150 (Stuhlmann et al. *Nat. Commun 2018*)
**** target peptide: LEVKEALNQDVNVPFANGI, Database number: #1348 RB1/D150 (Stuhlmann et al. *Nat. Commun 2018*)
***** target peptide: LYEGLPAEVREKMEEE, Database number: #1351 (Voss et al. *Science 2014*)
